# A restricted spectrum of missense *KMT2D* variants cause a multiple malformations disorder distinct from Kabuki syndrome

**DOI:** 10.1038/s41436-019-0743-3

**Published:** 2020-01-17

**Authors:** Sara Cuvertino, Verity Hartill, Alice Colyer, Terence Garner, Nisha Nair, Lihadh Al-Gazali, Natalie Canham, Victor Faundes, Frances Flinter, Jozef Hertecant, Muriel Holder-Espinasse, Brian Jackson, Sally Ann Lynch, Fatima Nadat, Vagheesh M. Narasimhan, Michelle Peckham, Robert Sellers, Marco Seri, Francesca Montanari, Laura Southgate, Gabriella Maria Squeo, Richard Trembath, David van Heel, Santina Venuto, Daniel Weisberg, Karen Stals, Sian Ellard, Genomics England Research Consortium, Anne Barton, Susan J. Kimber, Eamonn Sheridan, Giuseppe Merla, Adam Stevens, Colin A. Johnson, Siddharth Banka

**Affiliations:** 1 Division of Evolution and Genomic Sciences, School of Biological Sciences, Faculty of Biology, Medicine, and Health, The University of Manchester, Manchester, UK; 2 Division of Cell Matrix Biology and Regenerative Medicine, School of Biological Sciences, Faculty of Biology, Medicine, and Health, The University of Manchester, Manchester, UK; 3 Leeds Institute of Medical Research, Faculty of Medicine and Health, The University of Leeds, Leeds, UK; 4 Department of Clinical Genetics, Chapel Allerton Hospital, Leeds Teaching Hospitals Trust, Leeds, UK; 5 Astbury Centre for Structural Molecular Biology, Faculty of Biological Sciences, The University of Leeds, Leeds, UK; 6 Division of Developmental Biology & Medicine, School of Biological Sciences, Faculty of Biology, Medicine, and Health, The University of Manchester, Manchester, UK; 7 Centre of Genetics & Genomics Versus Arthritis, Manchester Academic Health Sciences Centre, The University of Manchester, Manchester, UK; 8 Department of Paediatrics, College of Medicine & Health Sciences, United Arab University, Al-Ain, UAE; 9 Liverpool Centre for Genomic Medicine, Liverpool Women’s NHS Foundation Trust, Liverpool, UK; 10 North West Thames Regional Genetics Service, Northwick Park Hospital, Harrow, UK; 11 Laboratorio de Genética y Enfermedades Metabólicas, Instituto de Nutrición y Tecnología de los Alimentos, Universidad de Chile, Santiago, Chile; 12 Department of Clinical Genetics, Guy’s & St Thomas NHS Foundation Trust, London, UK; 13 Department of Paediatrics, Tawam Hospital, Al-Ain, UAE; 14 Temple street Children’s University Hospital, Dublin, Ireland; 15 Wellcome Trust Sanger Institute, Cambridge, UK; 16 Medical Genetics Unit, St. Orsola-Malpighi, University of Bologna, Bologna, Italy; 17 Molecular and Clinical Sciences Research Institute, St George’s University of London, London, UK; 18 Department of Medical & Molecular Genetics, King’s College London, London, UK; 19 Division of Medical Genetics, Fondazione IRCCS Casa Sollievo della Sofferenza, San Giovanni Rotondo, Foggia, Italy; 20 Queen Mary University of London, London, UK; 21 Clinical Psychology Department, Royal Manchester Children’s Hospital, Manchester University Foundation NHS Trust, Health Innovation Manchester, Manchester, UK; 22 Molecular Genetics Department, Royal Devon and Exeter NHS Foundation Trust, Exeter, UK; 23 Institute of Biomedical and Clinical Science, University of Exeter Medical School, Exeter, UK; 24 Genomics England, London, UK; 25 Manchester Centre for Genomic Medicine, St. Mary’s Hospital, Manchester University Foundation NHS Trust, Health Innovation Manchester, Manchester, UK

**Keywords:** multiple congenital anomaly, Kabuki syndrome, KMT2D, histone 3 lysine 4 methyltransferase, intrinsically disordered region

## Abstract

**Purpose:**

To investigate if specific exon 38 or 39 *KMT2D* missense variants (MVs) cause a condition distinct from Kabuki syndrome type 1 (KS1).

**Methods:**

Multiple individuals, with MVs in exons 38 or 39 of *KMT2D* that encode a highly conserved region of 54 amino acids flanked by Val3527 and Lys3583, were identified and phenotyped. Functional tests were performed to study their pathogenicity and understand the disease mechanism.

**Results:**

The consistent clinical features of the affected individuals, from seven unrelated families, included choanal atresia, athelia or hypoplastic nipples, branchial sinus abnormalities, neck pits, lacrimal duct anomalies, hearing loss, external ear malformations, and thyroid abnormalities. None of the individuals had intellectual disability. The frequency of clinical features, objective software-based facial analysis metrics, and genome-wide peripheral blood DNA methylation patterns in these patients were significantly different from that of KS1. Circular dichroism spectroscopy indicated that these MVs perturb KMT2D secondary structure through an increased disordered to ɑ-helical transition.

**Conclusion:**

*KMT2D* MVs located in a specific region spanning exons 38 and 39 and affecting highly conserved residues cause a novel multiple malformations syndrome distinct from KS1. Unlike *KMT2D* haploinsufficiency in KS1, these MVs likely result in disease through a dominant negative mechanism.

## INTRODUCTION

Diverse developmental phenotypes resulting from distinct variants in the same gene are being increasingly recognized, largely due to wider application of next-generation sequencing and multicenter collaborations.^1^ These discoveries provide unique biological insights into gene and protein functions, and are critical for appropriate medical management, and counseling of patients and families.

**Fig. 1:**
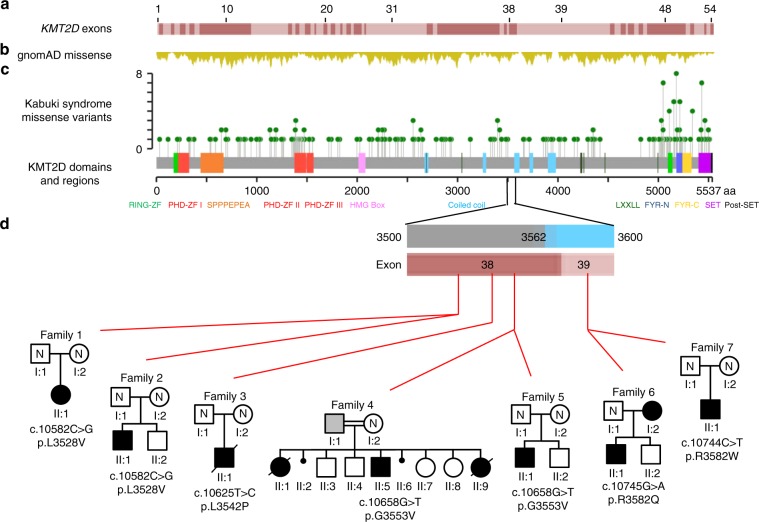
Affected individuals have missense variants in parts of exons 38 or 39 of *KMT2D*. We studied multiple affected individuals from seven families with missense *KMT2D* variants restricted to a region that encodes for 54 amino acids flanked by Val3527 and Lys3583 (ENST00000301067.7; NM_003482.3). (**a**) Schematic representation of *KMT2D* exons with each alternating exon represented in dark or light red shade (introns are not depicted). (**b**) Frequency of *KMT2D* missense variants (from gnomAD) in the general population is shown in yellow. Deeper troughs represent higher frequency at that particular location. (**c**) Green lollipop graph denoting the missense *KMT2D* variants in individuals with Kabuki syndrome from the published literature.^6^ The *y*-axis in this graph represents the frequency of the variant in the published literature. The *x*-axis is a schematic for the protein denoting the location of important domains and regions of KMT2D. Note that variants identified in this study are located in parts of exons 38 and 39 with high missense constraint but without any variants in individuals with Kabuki syndrome. (**d**) The region of interest of the *KMT2D* gene and protein in more detail. The red horizontal bar shows parts of exons 38 (amino acid 3503–3580) and 39 (amino acid 3581–4510). The blue vertical bars denote the coiled-coil regions. Red lines indicate the location of the variants identified in this study. Pedigree of each family is shown under the corresponding variant. Standard symbols are used to denote affected (filled symbols) and unaffected (unfilled) individuals. All individuals who were tested but not found to carry familial *KMT2D* variants are denoted by “N”. Father in family 4 (F4; I:1) was found to be likely mosaic and is denoted by gray square. In this family, genetic testing was not possible for the first born child (F4; II:1) but is shown as affected based on the clinical history.

Defective histone lysine methylation and chromatin remodeling defects underlie several congenital malformation disorders.^2^*KMT2D* encodes a very large (593 kDa) protein that catalyzes the mono-, di-, and trimethylation of the lysine 4 on histone 3 (H3K4) in a multiprotein complex.^3^ The KMT2D protein contains multiple known domains; however, the structure and function of several regions of the KMT2D protein remain unresolved. KMT2D also has a high level of predicted disorder content (55%) with many regions of intrinsic disorder, which are typical features of nuclear proteins that regulate transcription and chromatin organization.^4^ Kabuki syndrome type 1 (KS1, MIM 147920) is an autosomal dominant condition caused by loss-of-function *KMT2D* (MIM 602113) variants.^5^ Over 700 *KMT2D* variants have been reported in the literature in individuals with KS1. Approximately 80% of germline *KMT2D* variants causative for KS1 are predicted to be protein truncating and are thought to result in functional haploinsufficiency due to nonsense-mediated decay.^6^ In rare instances, individuals with KS1 have also been described to have a whole-gene deletion.^7^ Germline *KMT2D* missense variants (MVs) causative for KS1 cluster in certain functional regions of the protein, such as in the PHD fingers, RING-type zinc fingers, the FYR-N domain and the SET domain^6^ (Fig. 1a–c). Notably, germline MVs in some highly conserved regions of the protein have not yet been described in individuals with KS1.^6^ KS1 is characterized by neonatal hypotonia, feeding difficulties during infancy and early childhood, postnatal growth deficiency, skeletal anomalies, immune dysfunction, endocrine abnormalities, and congenital malformations of the heart, kidney, and palate.^8^ Almost all individuals with KS1 show global developmental delay and intellectual disability. Facial dysmorphism is the most distinguishing feature of the condition and is typically characterized by interrupted eyebrows, long palpebral fissures, eversion of the lateral part of the lower eyelids, large cupped ears, short columella, bulbous nasal tip, and pillowed lower lip.^8,9^ Here, we describe clustering of heterozygous *KMT2D* MVs, in a highly conserved central region of the protein, as causative of a novel phenotype with multiple malformations that is clinically and epigenetically distinct from KS1.

## MATERIALS AND METHODS

### Patient ascertainment, clinical characterization, and genetic studies

Ethical approval for molecular genetics research studies was obtained from the Research Ethics Committees of South Yorkshire (11/H1310/1), North West—Greater Manchester South (11/H1003/3), Cambridge South (10/H0305/83), the Republic of Ireland (GEN/284/12), Al-Ain Medical Human Research (ERH2015-3241-15-115), and East of England—Cambridge South (14/EE/1112). Informed consent for research studies was obtained from all participating families or individuals, as was permission to publish patient photographs. Ascertainment was driven by genotype in the first six families in whom *KMT2D* MVs of uncertain significance were identified by multigene panel testing or genome sequencing (GS) or exome sequencing (ES). The affected individual in family 7 was identified via targeted testing due to phenotypic similarity with other individuals described in this paper. All affected individuals underwent detailed clinical phenotyping (Table S1). Confirmation and segregation of all*KMT2D* variants was performed using conventional Sanger sequencing.

Total RNAs were extracted using RNeasy Mini kit (Qiagen) according to the manufacturer’s protocol and quantitative reverse transcription polymerase chain reaction (RT-qPCR) was performed using standard methods.

### Facial analysis

Ten previously unpublished photographs at different ages of six individuals in Ex38/39 *KMT2D* MVs cohort were uploaded onto and the results were obtained using the Face2Gene RESEARCH application (FDNA Inc., Boston, MA) for objective analysis of facial dysmorphology. Comparison was made with two groups of photographs of ten individuals, each with molecularly confirmed KS1 or CHARGE syndrome (OMIM 214800). The sensitivity and specificity of this classification system was analyzed by receiver operating characteristic (ROC) curves, and the corresponding area under the curve (AUC) and *p* values were calculated as described previously.^10^

### Peripheral blood DNA methylation

Peripheral blood was collected in EDTA tubes and genomic DNA was isolated using QIAamp DNA blood mini kit (Qiagen). Unmethylated cytosines were converted to uracil through bisulfite conversion using Zymo EZ DNA Methylation kit (Zymo Research). Genome-wide methylation was studied using Infinium MethylationEPIC BeadChip Kit (Illumina) (hereafter referred to as EPIC array) according to the manufacturer’s protocol. The array was stained and scanned using the Illumina iScan System. Data were processed using standard methods (Supplementary methods). Due to small number of samples and differences in ages of our patients and controls, we removed known age-associated probes.^11–13^ Principal component analysis (PCA) was used to visualize clustering of the samples based on differentially methylated positions (DMPs) between groups (*p* < 0.001). Overlap of DMPs between groups was assessed using InteractiVenn web based tool.^14^ Gene ontologies associated with DMPs were assessed using canonical pathway analysis (Ingenuity pathway analysis software, Qiagen, San Francisco, CA). Differentially methylated regions of the chromosome (DMRs) (>7 annotated CpGs) were analyzed on EPIC array data using the Bumphunter method^15^ in the Bioconductor package *ChAMP*.^16^

### H3K4me3 methylation

The pFlag-CMV2 FUSION–KMT2D construct^17^ was modified by inserting sequences for *KMT2D* that encode residues 3387–3697. Expression plasmids harboring *KMT2D* missense variants were generated by site-directed mutagenesis according to standard methods. HEK-293T cells were cultured in Dulbecco’s Modified Eagle Medium with 10% fetal bovine serum, penicillin (100 U/ml) and streptomycin (100 mg/ml) (Life Technologies). HEK-293T cells were transiently transfected using the polyethylenimine method, following published protocols.^18^ Cells were harvested 48 hours after transfection and used for protein extraction and histone methyltransferase assay. HEK-293T cells were plated in 12-well culture dishes at a density of 2.5 × 10^4^ cells/ml and then FLAG-KMT2D wild-type or mutated constructs were cotransfected together with an epigenetic reporter allele (“methyl reader”).^19^ Forty-eight hours after transfection, cells were washed and resuspended in 100 μl phosphate buffered saline (PBS) and subsequently plated into 96-well plates with black flat bottom (Corning®). The fluorescence signal was monitored using a Glomax 96 microplate luminometer (blue filter, Ex = 475 nm; Em = 500–550 nm) and was normalized to the methyl reader signal. Three or more biological replicates were performed for all assays.

### Protein analysis

Coiled-coil domains were predicted by MARCOIL, a hidden Markov model–based program that predicts the existence and location of potential coiled-coil domains in protein sequences.^20^ MultiCoil was used for predicting two- and three-stranded coiled coils.^21^ Gene synthesis of the wild-type and mutant sequences for *KMT2D* that encode residues 3231–3600 or 3503–3600 was carried out by GENEWIZ Inc. (South Plainfield, NJ). The sequences were subcloned into the bacterial expression vector pOPINF^22^ with a polyhistidine (6xHis) tag. Standard methods were followed for optimization of soluble recombinant protein expression at 12 **°**C in *E. coli* “ArcticExpress” cells (Agilent Technologies) and affinity purification with nickel-charged affinity resin columns (Ni-NTA Agarose) using fast protein liquid chromatography (ÄKTA Chromatography System, GE Healthcare Life Sciences). Protein purity was assessed by sodium dodecyl sulfate–polyacrylamide gel electrophoresis (SDS-PAGE) and western blotting. Purified proteins were buffer exchanged into 100 mM phosphate buffer, pH 7.2 (68.4 mM Na_2_HPO_4_, 31.6 mM NaH_2_PO_4_) and analyzed by circular dichroism (CD) spectroscopy, using a Chirascan Plus (Applied Photophysics) spectropolarimeter. Analyses of experimental protein CD spectra were by DichroWeb.^23^

## RESULTS

### Genetic studies

**Fig. 2:**
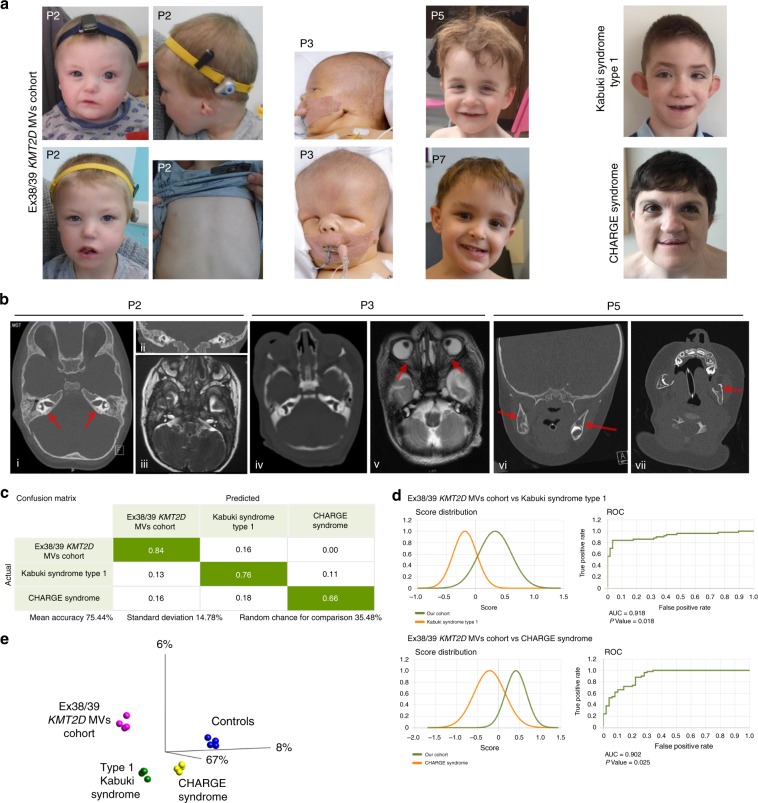
Missense *KMT2D* variants described in this study result in phenotype distinct from type 1 Kabuki and CHARGE syndromes. (**a**) Photographs of individuals described here with missense *KMT2D* variants. Note the wide range of facial features. P2, proband from family 2, is shown at two different ages. Note facial asymmetry, hypertelorism, bilateral epicanthic folds, bulbous nasal tip, downturned corners of the mouth, microtia, and hypoplastic nipples. P3, proband from family 3, has a box-shaped head, bilateral microphthalmia, severely hypoplastic left pinna, and ectopic left external auditory canal. P5 and P7, probands from families 5 and 7 respectively, have prominent forehead, broad nasal root, flat midface, and thin upper lip. P7’s eyebrows are laterally flared. One individual with Kabuki syndrome type 1 (KS1) is shown for comparison. Note arched eyebrows, long palpebral fissures, eversion of the lateral part of the lower eyelids, large cupped ears, short columella, bulbous nasal tip, and pillowed lower lip. In individual with CHARGE syndrome note hypertelorism, bulbous and large nasal tip, and a repaired cleft lip. (**b**) Computerized tomography (CT) (i and ii) and T2-weighted magnetic resonance imaging (MRI) (iii) of P2 demonstrating absence of the posterior part of the semicircular canals (red arrows) and normal anatomy of the lateral and anterior semicircular canals. Brain MRI of P3 (iv) to show well-formed right and left middle ear cavities, with the right cavity being smaller than the left. Bilaterally the cochlea, semicircular canals, and inner auditory canals appear normal. T1-weighted MRI to demonstrate a small left optic globe with ballooning of the optic disc bilaterally (red arrows) and optic disc colobomata (v). CT imaging of P5 (vi, vii) demonstrating the presence of cysts in the lower jaw (red arrows). (**c**) Face2Gene analysis with confusion matrix showing that the system is able to predict correctly each group with a mean accuracy of 75.44%. (**d**) Receiver operating characteristic (ROC) graphs show the probability curve where the area under the curve (AUC) (0–1) represents the measure of separability between two groups. Score distributions show the distribution of those probabilities. The higher the AUC, the better the model is at distinguishing between two groups. (**e**) Principal component analysis (PCA) shows the four groups analyzed in the DNA methylation array clustering separately (*p* < 0.001). Importantly, the samples from individuals described in this study cluster together and separate from those with type 1 Kabuki syndrome. *MV* missense variant.

We ascertained multiple individuals from seven families with missense variants restricted to a highly conserved region of 54 amino acids flanked by Val3527 and Lys3583 (ENST00000301067.7; NM_003482.3) (Figs. 1d and 2a, b; Fig. S1) (Supplementary case reports) (hereafter referred to as Ex38/39 *KMT2D* MVs). Identical de novo heterozygous exon 38 *KMT2D* c.10582C>G p.(Leu3528Val) variants was identified in probands of families 1 and 2. A de novo heterozygous exon 38 *KMT2D* c.10625T>C p.(Leu3542Pro) variant was identified in the proband of family 3. In family 4 (previously reported by Al-Gazali et al.^24^) and family 5 an identical heterozygous exon 38 *KMT2D* c.1658G>T p.(Gly3553Val) variant was seen in all affected individuals available for testing. In family 4, where multiple children were affected, the variant was shown to be mosaic in their father (Fig. S2) and in family 5 the variant had arisen de novo in the proband. In family 6, a heterozygous exon 39 *KMT2D* c.10745G>A p.(Arg3582Gln) variant was detected in the proband and his similarly affected mother. A de novo heterozygous exon 39*KMT2D* c.10744C>T p.(Arg3582Trp) variant, affecting the same residue that was substituted in family 6, was detected in family 7. Additional rare de novo or biallelic variants identified in our studies were all classed as likely benign (Table S2). All *KMT2D* MVs affected highly conserved residues (Fig. S1) and were absent in gnomAD, [Karczewski KJ, Francioli LC, Tiao G, et al. Variation across 141,456 human exomes and genomes reveals the spectrum of loss-of-function Q7 intolerance across human protein-coding genes. 2019] dbSNP,^25^ and ClinVar.^26^ The c.10744C>T (COSM431206) and c.10745G>A (COSM4604032) variants were found in COSMIC^27^ (Table S1). Overall, these results were highly indicative that the variants were pathogenic.

### Phenotype and facial analyses

As none of the patients were clinically diagnosed with KS, we compiled the clinical features of all affected individuals in detail (Fig. 2a, b) (Table 1). The most consistent clinical features of the affected individuals identified here included choanal atresia, athelia or hypoplastic nipples, branchial sinus abnormalities, neck pits, abnormalities of the lacrimal ducts, hearing loss, external ear malformations, and thyroid abnormalities or functional thyroid disease.

**Table 1:** Summary of clinical features of individuals with missense *KMT2D* variants.

#	Sex	Age at assessment	gDNA (hg19); Exon number; *KMT2D* cDNA; KMT2D protein	Inheritance	Physical anomalies and other phenotypes																					
					EE	HL	Ocu	Lac	Ch	Pal	Den	Br	Thy	Ma	Ca	GI	Ren	Gen	Imm	GR	FD	MD	SD	ID	MRI-B	Other comments
F1;II:1	F	13 years	12:49428008G>C; ex 38; c.10582C>G; p.(Leu3528Val)	DN	Y	Y	Y	Y	Y	N	N	Y	Y	Y	N	N	N	N	N	Y	N	N	Y	N	NK	Moderate thoracic scoliosis; clinical suspicion of CHARGE syndrome
F2;II:1	M	2 years 8 months	12:49428008G>C; ex 38; c.10582C>G; p.(Leu3528Val)	DN	Y	Y	N	Y	Y	N	Y	Y	N	Y	N	Y	N	N	N	Y	Y	N	Y	N	Y	None
F3;II:1	M	28 days	12:49427965A>G; ex 38; c.10625T>C; p.(Leu3542Pro)	DN	Y	N	Y	N	Y	N	NA	Y	N	N	Y	N	N	N	NA	NA	Y	NA	NA	NA	N	Died at 28 days of age
F4;II:5	M	9 years	12:49427932C>A; ex 38; c.10658G>T; p. (Gly3553Val) (variant was mosaic in I:1)	Pat	N	Y	N	Y	Y	N	Y	Y	Y	Y	Y	Y	N	Y	Y	Y	N	N	Y	N	N	None
F4;II:9	F	4 months		Pat	N	Y	N	Y	Y	N	N	Y	Y	Y	Y	N	N	N	Y	Y	N	NA	NA	NA	N	Died at 4 months of age following pneumonia
F4;I:1	M	39 years		NK	N	N	N	N	N	N	N	N	N	N	N	N	N	N	N	N	N	N	N	N	N	None
F5;II:1	M	3.5 years	12:49427932C>A; ex 38; c.10658G>T;p. (Gly3553Val)	DN	Y	Y	N	N	Y	N	Y	Y	Y	Y	Y	Y	N	N	Y	Y	Y	Y	Y	N	Y	None
F6;II:1	M	6 years	12:49427743C>T; ex 38; c.10745G>A;p.(Arg3582Gln)	Mat	Y	Y	Y	Y	N	Y	N	N	Y	N	N	N	N	N	N	N	N	N	Y	N	N	Clinical suspicion of branchiootorenal syndrome
F6;I:2	F	35 years		NK	Y	Y	N	Y	N	Y	N	N	N	N	N	N	N	N	N	N	N	N	N	N	N	Clinical suspicion of branchiootorenal syndrome
F7;II:1	M	3 years 5 months	12:49427744G>A; ex 39; c.10744C>T; p.(Arg3582Trp)	DN	N	Y	N	Y	Y	N	N	Y	Y	Y	N	N	N	Y	Y	N	Y	N	N	N	N	Clinical suspicion of branchiootorenal syndrome

Individuals’ identification number correlates with the pedigrees in Fig. 1. Full clinical details are provided in Supplementary Table S1.

*Br*   branchial, *Ca *  cardiac, *cDNA* complementary DNA, *Ch *  choanal, *Den *  dental, *DN* de novo, *EE *  external ears,*F *  family, *FD *  feeding difficulties, *Gen*   genitalia, *gDNA* genomic DNA, *GI *  gastrointestinal, *GR *  growth retardation, *HL *  hearing loss, *Imm*  immune system, *ID *  intellectual or learning disability, *Lac *  lacrimal,*Ma *  mammary, *Mat *  maternal, *MD *  motor delay, *MRI-B*   magnetic resonance imaging of brain,*N*   No anomaly or abnormality, *NA *  not applicable,*NK *  not known, *Ocu *  ocular, *Pat *  paternal, *Pal *  palatal, *Ren*  renal, *SD* speech delay, *Thy *  thyroid,*Y*   yes anomaly or abnormality present.

To examine if the phenotype of the affected individuals is distinct from that of KS1, we compared the frequencies of major phenotypes observed in Ex38/39 *KMT2D* MVs cohort and in individuals with KS1 (Table 2). Only hearing loss and external ear abnormalities were consistent major features of both conditions. In contrast, cleft lip or palate, renal structural abnormalities, and seizures are common in KS1 but were absent in the Ex38/39 *KMT2D* MVs cohort. Due to the clinical overlap, we also compared the frequencies of phenotypes observed in the Ex38/39 *KMT2D* MVs cohort with individuals with CHARGE syndrome. This comparison also showed several differences (Table 2). External ear malformations, although seen in all three groups are of different nature (small or absent external ears in the Ex38/39 *KMT2D* MVs cohort, large prominent ears in KS1, and simple dysplastic in CHARGE syndrome). Markedly, intellectual disability that is almost universal in KS1 and CHARGE syndromes was absent in the Ex38/39 *KMT2D* MVs cohort. As facial dysmorphism is the most specific and sensitive feature of KS1,^8,9^ we performed computer-based objective analyses based on syndrome-specific classifiers of facial features. This showed that the facial features of individuals in the Ex38/39 *KMT2D* MVs cohort were significantly different to those with KS1 (AUC = 0.918; *p* = 0.018) and CHARGE syndrome (AUC = 0.902; *p* = 0.025) (Fig. 2c, d). Overall, these analyses were highly suggestive that the phenotype of the individuals described in this study is distinct from that of Kabuki and CHARGE syndromes.

**Table 2:** Comparison of the phenotype between our cohort and type 1 Kabuki and CHARGE syndromes.

Feature	Ex38/39 KMT2D MVs	Type 1 Kabuki syndrome	CHARGE syndrome
Branchial sinus/neck pits	7/9 (78%)	Not reported or extremely rare	Not reported or extremely rare
Hearing loss	8/9 (89%)	Common	Common
External ear abnormalities	6/9 (67%) (small, hypoplastic, or absent)	Common (usually prominent and simple)	Common (usually simple or dysplastic)
Structural abnormality of eye	2/9 (22%)	Rare	Common
Abnormality of lacrimal ducts	7/9 (78%)	Rare	Rare
Choanal atresia	7/9 (78%)	Rare	Common
Cleft lip/palate	0	Common	Common
Athelia/hypoplastic nipples	6/9 (67%)	Not reported (prominent breasts are common)	Rare
Congenital heart disease	3/9 (33%)	Common	Common
Renal structural abnormality	0	Common	Common
Seizures	0	Common	Common
Intellectual disability	0	Common	Common
Feeding difficulties	5/9 (44%)	Common	Common
Short stature	5/9 (56%)	Common	Common
Thyroid abnormality/hypothyroidism	6/9 (67%)	Rare	Rare
Abnormality of immune system/recurrent infections	4/9 (44%)	Common	Common

This table compares the clinical features of our cohort of individuals with missense *KMT2D* variants with type 1 Kabuki and CHARGE syndromes. We have considered common and rare features as those that occur in >25% and <25% of affected individuals, respectively. For the Ex38/39 KMT2D MVs cohort we have excluded the individual (F4; I:1) with mosaic variant to calculate the frequencies of the clinical features.

### Peripheral blood DNA methylation

Since DNA methylation signatures can differentiate pathogenic and nonpathogenic *KMT2D* variants,^28^ we examined if the epigenetic profile of the condition we described herein is distinct from that of KS1. We compared the DNA methylation patterns in peripheral blood collected from the following individuals: four affected individuals from the Ex38/39 *KMT2D* MVs cohort, four individuals with KS1, four with CHARGE syndrome, and four controls (Table S3). We validated our results by comparing them with the hypo- and hypermethylated CpG sites in individuals with KS1 listed by Butcher et al. (Fig. S3).^28^ The PCA analysis of the DNA methylation profiles of samples from the Ex38/39 *KMT2D* MVs cohort clustered together and differed from those of controls, KS1, and CHARGE syndrome (Fig. 2e). Intersection analysis showed that the samples from the Ex38/39 *KMT2D* MVs cohort and KS1 share only 915 differentially methylated CpG sites (*p* < 0.001) (Fig. S4). These results were highly suggestive that Ex38/39 *KMT2D* MVs result in a condition that is epigenetically distinct from both Kabuki and CHARGE syndromes.

### RT-qPCR

To explore the disease mechanism we studied the effect of these variants on the expression of *KMT2D*. Fibroblasts were obtained from two affected individuals from our cohort (proband from family 7 and family 3) (Table S3) and one control. Using RT-qPCR, we observed no significant differences in the level of *KMT2D* expression in fibroblasts of affected individuals compared with the control (Fig. S5).

### Histone H3K4 methylation assays

To experimentally test the functional impact of *KMT2D* missense variants, we generated FLAG-tagged versions of six *KMT2D* missense alleles: p.Leu3528Val, p.Leu3542Pro, p.Gly3553Val, p.Gln3575His,^29^ p.Arg3582Trp, and p.Arg3582Gln. Fluorescence data revealed no changes in trimethylation H3K4 levels between all the missense variants tested in this assay compared with*KMT2D* normal control activity (Fig. S6).

### Protein analysis

**Fig. 3:**
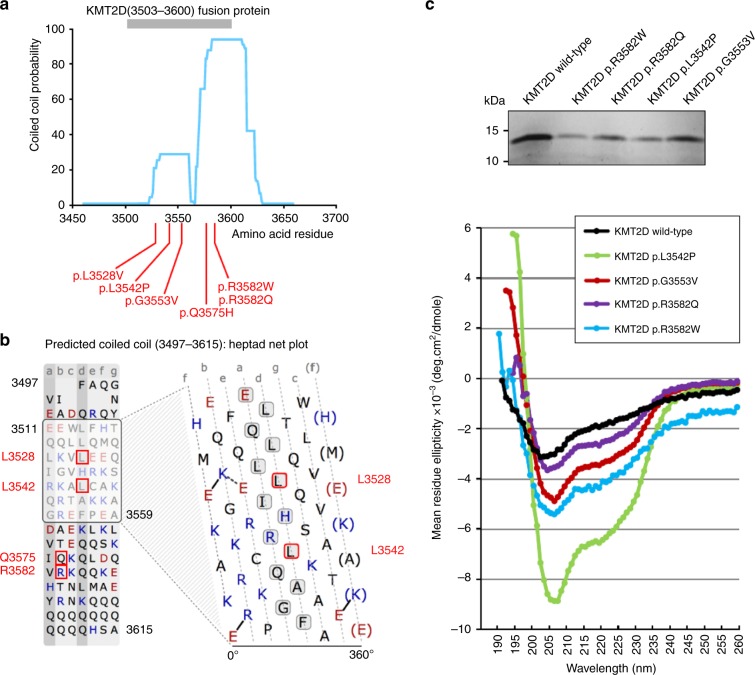
Missense variants described in this study perturb protein secondary structure in KMT2D recombinant proteins. (**a**) Central, highly conserved region of KMT2D containing a coiled-coil domains predicted by MARCOIL (blue trace). All *KMT2D* missense variants described here (red lines) occur within or close to the predicted coiled-coil domain (residues 3562–3614). KMT2D fusion proteins (residues 3503–3600; gray bar) contained the missense variants described here. (**b**) Heptad net plot view of a potential coiled-coil domain, predicted by MARCOIL between residues 3511 and 3559 of KMT2D, showing approximately seven heptad repeats of hydrophobic or nonpolar residues (black letters) and charged residues (blue or red letters). Missense variants of residues within or close to the predicted coiled-coil domain are indicated by red boxes. Positions of residues within the predicted heptad repeat sequences are labeled a to g. Residues at the “a” and “d” positions (gray boxes), which include Leu3528 and Leu3542, form the hydrophobic seam in a coiled coil. (**c**) Upper panel: expression of wild-type and mutant recombinant KMT2D fusion proteins, as indicated. Lower panel: circular dichroism (CD) spectroscopy traces of recombinant KMT2D wild-type and mutant proteins showing moderate levels of disordered secondary structure in the wild-type protein (black trace) with perturbed secondary structure and higher proportions of ɑ-helical structure in all recombinant KMT2D proteins with missense variants (purple, red, light blue, and green traces).

As the variants did not appear to impact on *KMT2D* expression or direct H3K4 trimethylation activity, we considered the possibility that variants affected protein–protein interactions with KMT2D. The variants identified in this study do not occur in a known functional domain of the protein, but are instead in a central, highly conserved region that also contains short polyQ tracts (Fig. S1). All of the variants occur within or close to a coiled-coil domain (residues 3562–3614; Fig. 3a) predicted by MARCOIL.^20^ Furthermore, MultiCoil^21^ identified heptad repeat sequences that mediate a possible coiled-coil dimer between residues 3506 and 3610 (heptad net plot, Fig. 3b). Analysis of the CD spectrum of the KMT2D wild-type protein fragment (3503–3600 residues, molecular weight 11.8 kDa) by DichroWeb^23^ revealed the following predicted proportions of secondary structure: 0.14 α-helix, 0.26 β-strand, 0.27 turn, and 0.32 disordered. A larger KMT2D wild-type protein fragment (3231–3600 residues, molecular weight 40.7 kDa) had the following proportions: 0.22 α-helix, 0.18 β-strand, 0.19 turn, and 0.41 disordered (Fig. S7). All recombinant KMT2D proteins with missense variants had perturbed secondary structure with decreased disorder and increased proportions of predicted α-helical structure (Fig. 3c).

### Differentially methylated region analysis

We used the data generated from our peripheral blood DNA methylation experiments to explore the likely dysregulated pathways. Within the 5162 differentially methylated CpG sites in Ex38/39 *KMT2D* MVs cohort compared with control samples, we observed significant enrichment for CpG sites corresponding to genes related to morphology of the head, embryonic development, cell proliferation, and development of body axis (Fig. S4). Next, we investigated the potential disease mechanism by analyzing DMRs in the Ex38/39 *KMT2D* MVs cohort compared with control samples. We identified 83 such DMRs including several genes associated with clinical features, such as hearing impairment or hypothyroidism, that we observed in the Ex38/39 *KMT2D* MVs cohort (Table S4) (Fig. S4).

## DISCUSSION

Multiple lines of evidence suggest that the Ex38/39 *KMT2D* MVs identified in this study are the cause of the phenotypes of the affected individuals. These include (1) the de novo occurrence of the variants in five families (Fig. 1), (2) segregation of the variants with the phenotype in a multiplex family (family 6), (3) presence of variant in mosaic state in an unaffected parent of another multiplex family (family 4), (4) absence of these variants from control databases, (5) identical variants in families 1 and 2 (p.Leu3528Val) and in families 4 and 5 (p.Glu3553Val), (6) distinct variants affecting the same codon in families 6 and 7 (p.Arg3582Glu) and (p.Arg3582Trp), (7) clustering of the variants within a span of just 54 amino acids in a protein of size 5537 amino acids with very strong evolutionary conservation of all affected residues (Fig. S1), (8) strong similarity of the phenotype in families 1–6 that were ascertained on the basis of genotype, and (9) identification of the variant in family 7 through targeted single-gene phenotype-led testing (Table 1 and S1) (Fig. 2).

The affected individuals we describe share some features with KS1, including hearing loss, dental anomalies, feeding difficulties, failure to thrive, and abnormalities of the immune system. However, the most commonly observed malformations in individuals with KS1, including cleft palate, congenital heart disease, and renal anomalies, were infrequent or absent in the Ex38/39 *KMT2D* MVs cohort (Table 2).^8^ Importantly, the affected individuals in our study did not display intellectual disability or hypotonia, which are almost universal features in all patients with KS1. Speech delay noted in several individuals was attributable to their hearing impairment. Furthermore, the typical dysmorphic features of KS1 were not seen in the cohort described here.^8^ This was supported by the results of our objective facial analysis studies (Fig. 2). We found that the DNA methylation pattern of individuals described here was distinct from what has been described in KS1. Notably, the DNA methylation studies were performed in a small number of individuals and testing in a larger patient cohort will be required to validate our findings. Nevertheless, collectively these results strongly suggest that the restricted spectrum of *KMT2D* missense variants in exon 38 and 39 identified in this study result in a multiple malformation disorder that is genetically, phenotypically, and epigenetically distinct from KS1.

The variants described here are flanked by Val3527 and Lys3583. However, it remains to be seen if variants outside this window may also result in the same phenotype. Furthermore, the phenotype spectrum of individuals described here is very wide, ranging from early death of three individuals (from families 3 and 4) to a relatively mild phenotype (e.g., the affected mother in family 6). This may be due to allelic heterogeneity or other genetic, epigenetic, or environmental factors. The identification of larger cohorts of individuals will be required to resolve these questions.

Some features of the Ex38/39 *KMT2D* MVs cohort overlap with those of CHARGE syndrome (OMIM 214800), including coloboma, choanal atresia, and ear anomalies (Table S5).^30^ However, none of the affected individuals fulfill the clinical diagnostic criteria for CHARGE syndrome.^30^ Molecularly, KMT2D and CHD7 both interact with members of the WAR complex^31^ and clinical overlap between *KMT2D* variants and CHARGE syndrome has been previously postulated.^29,31^ Two of these previous reports described individuals with clinical features that match those of the Ex38/39 *KMT2D* MVs cohort and that carry *KMT2D* missense variants, one with p.Gln3575His and another with p.Leu3564Val, that lie within the central conserved KMT2D region we describe.^29,32^ We propose that these previously published individuals have neither KS1 nor CHARGE syndrome, and fit better with the condition that we describe in this study. Other disorders that phenotypically overlap with the condition described here include branchiootorenal syndrome (BORS, OMIM 113650), branchiootic syndrome 2 (OMIM 120502), branchiooculofacial syndrome (OMIM 113620), and Bamforth–Lazarus syndrome (OMIM 241850) (Table S5).

Athelia or hypoplasia of the nipples is a rare clinical feature of unknown incidence and was present in more than half the patients in our report. On the contrary, most individuals with KS1 tend to develop prominent breasts during infancy and early childhood. A single case report has described an association between hypoplastic nipples and CHARGE syndrome.^33^ The combination of athelia and choanal atresia is an even rarer association, previously only described in carbimazole embryopathy.^34^ In the absence of an antenatal history of maternal carbimazole use, this combination of features would be very suggestive of the condition described in this paper. Of note, carbimazole is a pro-drug that is converted to methimazole, which inhibits thyroid peroxidase. We speculate that the*KMT2D* variants described here perhaps directly or indirectly result in dysfunction, downregulation, or inhibition of thyroid peroxidase or developmental pathways involving this enzyme. On review of the literature with a focus on the combination of choanal atresia and athelia, we identified two previous case reports describing features very similar to the families described here including athelia, choanal atresia, failure to thrive, congenital cardiac disease, and preauricular pits.^35,36^ Due to the clinical similarities, we suspect that our report provides a possible explanation for the etiology of disease in these historical cases.

In contrast to the observations for individuals with Kabuki-causing*KMT2D* missense variants,^17^ the H3K4 methyltransferase activities of the variants described here were normal (Fig. S6). Notably, KMT2D also possesses H3K4 me1 and me2 methyltransferase activity that we have not tested. However, given the genetic and clinical findings, it appears unlikely that the phenotype results simply from the loss of H3K4 methyltransferase function. Importantly, the variants identified in this study are in a highly conserved central region that contains predicted coiled-coil domains (Fig. 1b) as well as short polyQ tracts (Fig. S1). The polyQ tracts at the C-terminus end of the highly conserved central region are likely to be unstructured and to destabilize coiled-coil formation, potentially with the formation of intrinsically disordered regions of secondary protein structure. The results of our CD experiments (Fig. 3) suggest that the central region of KMT2D is indeed disordered, which is consistent with the usual major roles of intrinsically disordered regions in mediating and modulating protein–protein interactions.^4,37^ However, pathogenic missense variants in the central region, as in the Ex38/39 *KMT2D* MVs cohort, could prevent normal transitions between disordered and ɑ-helical structure in a coiled-coil domain, or reduce the levels of intrinsic disorder, thereby disrupting normal specific protein–protein interactions in the KMT2D chromatin remodeling complex.

In the peripheral blood of affected individuals one of the most significant hypomethylated DMRs that we observed encompasses the transcription start site (TSS) region of *HOXA2* (OMIM 604685), variants in which cause autosomal dominant microtia with or without hearing impairment (612290) (Fig. S4). *HOXA2* is a fundamental transcriptional factor that regulates *EYA1* (linked with BORS) and orchestrates morphogenesis of the auricle. Notably, ear anomalies were seen very frequently in the Ex38/39 *KMT2D* MVs cohort. We also identified hypermethylation in the gene body of *PAX8* (OMIM 167415), variants in which cause congenital hypothyroidism due to thyroid dysgenesis or hypoplasia (OMIM 218700) (Fig. S4). Hypothyroidism was a frequent feature in the Ex38/39 *KMT2D* MVs cohort. Furthermore, a few CpG sites corresponding to genes involved in neural crest development showed altered DNA methylation pattern. In particular, we observed hypomethylation in the regions corresponding to *ZIC3* (OMIM 300205), *TFAP2E* (OMIM 614428), and *SOX10* (OMIM 602229) and a hypermethylation in the region corresponding to *ZEB2* (OMIM 605802). All these regulators influence neural crest migration.^38^ Although the expression of these genes requires to be tested in relevant tissue, our results suggest that the variants described in this study might be deleterious to the normal expression or function of key transcription factors that might impair specific developmental processes.

In summary, we describe a variable disorder caused by missense variants in parts of exons 38 and 39 of *KMT2D* that is clinically, genetically, and epigenetically distinct from KS1. This condition is characterized by anomalies of the branchial arch, ears, choanae, lacrimal ducts, nipples, thyroid, growth, and the immune system. This work provides insights into the diverse roles of KMT2D in embryonic development and uncovers the importance of a previously unstudied region of this critical protein that is likely to be an intrinsically disordered region that mediates and modulates protein–protein interactions. Further work will be required to determine the link between the*KMT2D* variants described here and the human and cellular phenotypes observed. Overall, this work emphasizes the possibility of undiscovered phenotypes linked with even well-studied genes.^39,40^

## Supplementary information

Supplementary Material

## Web resources

www.agilent.com/genomics/suredesign

https://app.face2gene.com/research
