# Correction: A restricted spectrum of missense *KMT2D* variants cause a multiple malformations disorder distinct from Kabuki syndrome

**DOI:** 10.1038/s41436-020-0784-7

**Published:** 2020-03-23

**Authors:** Sara Cuvertino, Verity Hartill, Alice Colyer, Terence Garner, Nisha Nair, Lihadh Al-Gazali, Natalie Canham, Victor Faundes, Frances Flinter, Jozef Hertecant, Muriel Holder-Espinasse, Brian Jackson, Sally Ann Lynch, Fatima Nadat, Vagheesh M. Narasimhan, Michelle Peckham, Robert Sellers, Marco Seri, Francesca Montanari, Laura Southgate, Gabriella Maria Squeo, Richard Trembath, David van Heel, Santina Venuto, Daniel Weisberg, Karen Stals, Sian Ellard, Genomics England Research Consortium, Anne Barton, Susan J. Kimber, Eamonn Sheridan, Giuseppe Merla, Adam Stevens, Colin A. Johnson, Siddharth Banka

**Affiliations:** 1 Division of Evolution and Genomic Sciences, School of Biological Sciences, Faculty of Biology, Medicine, and Health, The University of Manchester, Manchester, UK; 2 Division of Cell Matrix Biology and Regenerative Medicine, School of Biological Sciences, Faculty of Biology, Medicine, and Health, The University of Manchester, Manchester, UK; 3 Leeds Institute of Medical Research, Faculty of Medicine and Health, The University of Leeds, Leeds, UK; 4 Department of Clinical Genetics, Chapel Allerton Hospital, Leeds Teaching Hospitals Trust, Leeds, UK; 5 Astbury Centre for Structural Molecular Biology, Faculty of Biological Sciences, The University of Leeds, Leeds, UK; 6 Division of Developmental Biology & Medicine, School of Biological Sciences, Faculty of Biology, Medicine, and Health, The University of Manchester, Manchester, UK; 7 Centre of Genetics & Genomics Versus Arthritis, Manchester Academic Health Sciences Centre, The University of Manchester, Manchester, UK; 8 Department of Paediatrics, College of Medicine & Health Sciences, United Arab University, Al-Ain, UAE; 9 Liverpool Centre for Genomic Medicine, Liverpool Women’s NHS Foundation Trust, Liverpool, UK; 10 North West Thames Regional Genetics Service, Northwick Park Hospital, Harrow, UK; 11 Laboratorio de Genética y Enfermedades Metabólicas, Instituto de Nutrición y Tecnología de los Alimentos, Universidad de Chile, Santiago, Chile; 12 Department of Clinical Genetics, Guy’s & St Thomas NHS Foundation Trust, London, UK; 13 Department of Paediatrics, Tawam Hospital, Al-Ain, UAE; 14 Temple street Children’s University Hospital, Dublin, Ireland; 15 Wellcome Trust Sanger Institute, Cambridge, UK; 16 Medical Genetics Unit, St. Orsola-Malpighi, University of Bologna, Bologna, Italy; 17 Molecular and Clinical Sciences Research Institute, St George’s University of London, London, UK; 18 Department of Medical & Molecular Genetics, King’s College London, London, UK; 19 Division of Medical Genetics, Fondazione IRCCS Casa Sollievo della Sofferenza, San Giovanni Rotondo, Foggia, Italy; 20 Queen Mary University of London, London, UK; 21 Clinical Psychology Department, Royal Manchester Children’s Hospital, Manchester University Foundation NHS Trust, Health Innovation Manchester, Manchester, UK; 22 Molecular Genetics Department, Royal Devon and Exeter NHS Foundation Trust, Exeter, UK; 23 Institute of Biomedical and Clinical Science, University of Exeter Medical School, Exeter, UK; 24 Genomics England, London, UK; 25 Manchester Centre for Genomic Medicine, St. Mary’s Hospital, Manchester University Foundation NHS Trust, Health Innovation Manchester, Manchester, UK

Correction to: *Genetics in Medicine* **22**:2020 10.1038/s41436-019-0743-3 published online 17 January 2020.

An incorrect reference was cited in the fourth sentence of the Results section. The correct citation should have been Al-Gazali LI, Hamid Z, Hertecant J et al. An autosomal recessive syndrome of choanal atresia, hypothelia/athelia and thyroid gland anomalies overlapping Bamforth syndrome, ANOTHER syndrome and methimazole embryopathy. Clin Dysmorphol 2002;2:79–85. This has now been corrected in both the PDF and HTML versions of the Article.

