# Supplementary material for: A restricted spectrum of missense KMT2D variants cause a multiple malformations disorder distinct from Kabuki syndrome
Source: Genet Med. 2020 Jan 17;22(5):867–77. doi: 10.1038/s41436-019-0743-3 (PMC7200597; doi:10.1038/s41436-019-0743-3)
Supplement: Supplementary file 1 — Supplementary Material [file 41436_2019_743_MOESM1_ESM.docx]

# Supplemental material

## Case reports

**Family 1:** Proband is a 13 year old female born to non-consanguineous parents (Figure 1) (Table 1 and S1). Echogenic bowel and increased uterine artery Doppler pulsatility were detected on antenatal scans at 20 weeks. She was born via vaginal delivery at 34 weeks of gestation with weight of 1.790 kg (-0.92 SD), length of 44 cm (-0.22 SD) and head circumference of 31 cm (-0.13 SD). Bilateral choanal atresia, right incomplete iridal coloboma and bilateral athelia were noticed after birth. Unilateral narrow naso-lacrimal duct was found on a facial bone CT scan. She achieved motor milestones within the normal range. Expressive speech developed soon after bilateral hearing aids were placed at the age of 1 year for severe sensorineural hearing loss. At 9 years of age, hypothyroidism was diagnosed. At 10 years of age, growth hormone replacement therapy was started for short stature (height <10^th^ percentile; target height >25^th^ percentile) and height velocity deceleration (-1.49 SD), although there was no evidence of GH deficiency. Heart and abdomen ultrasound scans revealed no abnormalities. She has mild dolichocephaly, tall forehead, sparse eyebrows, down-slanted palpebral fissures, depressed nasal bridge, low hanging columella, broad philtrum, low-set ears with underdeveloped and overfolded helix, auricular pits, thin upper lip, moderate micrognathia and bilateral branchial arch fistulas. She also has bilateral single palmar creases and moderate thoracic scoliosis. Array comparative genomic hybridisation (a-CGH) revealed a paternally inherited Xp22.33 microduplication, including *SHOX* (OMIM 312865). A customized HaloPlex Target Enrichment panel (Agilent Technologies, Inc., Santa Clara, CA) designed using Agilent’s SureDesign tool was performed with a clinical suspicion of a chromatin remodelling disorder and revealed a heterozygous exon 38 *KMT2D* c.10582C>G (p.(Leu3528Val)) variant. No other significant variants were identified and targeted parental testing confirmed the *KMT2D* variant to be *de novo.* Affected individual from Family 1 was sequenced on a clinical basis by a customized.

**Family 2:** Proband is a 4 year and 7 months old boy born to non-consanguineous parents (Figures 1 and 2A) (Table 1 and S1). He was born at 37 weeks of pregnancy. He was found to have left sided lower motor neuron facial palsy, left sided choanal atresia requiring multiple dilatations, blocked lacrimal duct, bilateral microtia with sinuses discharging intermittently, bilateral moderate mixed sensorial neural and conductive hearing loss mild micrognathia, absent left nipple, and gastro-oesophageal reflux. He had mild speech and language delay. His eight primary teeth were extracted due to severe caries. He has a history of repeated respiratory tract infections. His MRI brain scan performed at the age of 18 months showed bilateral posterior semi-circular canal hypoplasia with normally formed cochlear and vestibule (Figure 2B). The facial nerves were very slender, but facial colliculus was seen. He has facial asymmetry, hypertelorism, bilateral epicanthic folds, a bulbous tip of the nose, down-turned corners of the mouth and bilateral microtia. Trio whole genome sequencing (WGS) was performed as part of the 100,000 Genomes project ^1^.identified a heterozygous *de novo* exon 38 *KMT2D* c.10582C>G (p.(Leu3528Val)) variant.

**Family 3:** Proband was the first child, a male, born to non-consanguineous parents (Figures 1 and 2A) (Tables 1 and S1). No anomaly scan had been performed earlier in pregnancy. Polyhydramnios and intra-uterine growth retardation (IUGR) were noted at 39 weeks of gestation. He was born at 40 weeks and 3 days of gestation with a weight 2.62Kg (-1.99 SD) and head circumference of 37cms (1.41 SD). Bilateral severe microphthalmia were noted after birth. MRI brain revealed optic disc colobomata, three on the left and one on the right side (Figure 2B). Bilateral ballooning of the ocular lenses was noted. He also had bilateral choanal atresia. The vomer was broad posteriorly and there was significant bony choanal stenosis. Bilateral membranes were seen extending across the narrowed choana. Maxillary sinuses were not formed. He had bilateral microtia with sinus tracts opening to the lateral side of the face. The left pinna was severely hypoplastic and was adjacent to an ectopic position of the left external auditory canal. It opened posterior to the left mandibular ramus, in the anterior triangle of the neck. It extended via a broad channel to the anterior aspect of the left middle ear cavity and also extended deep to the oropharynx in a fistulous channel of moderate calibre. A deep pit was visualised posterior to the right mandibular ramus in the anterior triangle of the neck which could represent an atretic ectopic external auditory canal. CT scan of skull and facial bones revealed soft tissue atresia of the right auditory canal. Bilaterally, the malleus and incus appeared to be closely opposed to the tegmentum and may have been fixed. The stapes was not identified on either side. The cochlea, the semicircular canals and the inner auditory canals appeared normal. The child was admitted to the Intensive Care Unit for ventilatory support and died on day 34. Blood and skin karyotype and a-CGH were normal. Trio WES performed using Agilent Sure Select Human All Exon Kit v6/Illumina NextSeq identified a heterozygous *de novo* exon 38 *KMT2D* c.10625T>C (p.(Leu3542Pro)) variant.

**Family 4:** This family was previously reported by Al-Gazali et al., 2002. The parents are first cousins of Palestinian origin. Their first child was a female (II:1 in Family 4 in Figure 1) who was born with choanal atresia and had died shortly after birth. Further medical details of this child are unavailable. The couple had two more similarly affected, and four other unaffected children.

One of these affected children, a male (II:5 in Family 4 in Figure 1) (Tables 1 and S1), was reviewed at the age of 10 years and was found to have right choanal atresia, bilateral absent lacrimal ducts, bilateral conductive hearing loss, a small thyroid gland, hypoplastic nipples, a patent foramen ovale and undescended left testis. His height and weight were <5th centile. He was reported to suffer from chronic respiratory infections in the first year of life, which lead to chronic interstitial lung fibrosis and right ventricular hypertrophy. He had sparse hair and eyebrows, dystrophic nails, down-slanting palpebral fissures, low set ears and hypoplastic alai nasi. Cranial CT scan, barium swallow and renal ultrasound scan were normal. Thyroid function tests were also within the normal range. There was not history of intellectual disability.

Another affected child, a female (II:9 in Family 4 in Figure 1) (Tables 1 and S1), had a birth weight <10th centile. She was noted to have widely spaced eyes, a depressed nasal bridge, a low right ear and a neck pit. She had hypoplastic nipples and bilateral choanal atresia. She had congenital hypothyroidism secondary to complete absence of the thyroid gland. She had primary pulmonary hypertension. Skeletal survey, abdominal ultrasound scan and brain CT were normal. Her length and weight were <3^rd^ centile at 3 months of age. She died at the age of 4 months following a respiratory infection.

WES was performed In as described previously^2^ on three members of this family, the affected female child (II:9) and both unaffected parents, on DNA samples extracted from peripheral blood. No rare *de novo* variants were identified. Five rare bi-allelic variants were recognised, none of which were thought to be linked to the phenotype (Table S2). Additionally, a heterozygous exon 38 *KMT2D* c.1658G>T (p.(Gly3553Val)), was identified in the affected child and in her father but was absent in the mother. Reads for each call at this position were 30 reference to 33 alternative allele in the affected girl, and 33 reference to 17 alternative allele in the father, raising the possibility of paternal mosaicism. Sanger sequencing of the paternal DNA sample showed a smaller peak for the *KMT2D* c.1658G>T variant in comparison with the reference allele confirming mosaic status of the variant in his blood (Figure S1). Further tissue samples from the father were not available for testing. Sanger sequencing confirmed the presence of variant in the proband (II:9) and in her affected brother (II:5). DNA samples from the deceased sibling (II:1) and the other unaffected siblings were unavailable.

**Family 5:** The proband is 3 year and 6 months old boy is one of two children born to non-consanguineous parents (Figures 1 and 2A) (Table 1 and S1). Antenatal scans had identified increased nuchal thickness and echogenic bowel identified at the 12 and 20 week respectively. Prenatal investigations showed a normal karyotype and absence of the common *CFTR* (OMIM) variants. He was born via an induced vaginal delivery at 37 weeks of gestation with a weight of 2.35kg (-1.31 SD). Bilateral choanal atresia, bilateral athelia, branchial cysts, tongue tie, hypothyroidism, absent thymus, absent gall bladder, a caudothalamic cyst, preauricular pits, atrial septal defect and neonatal teeth were noticed soon after birth.  He sat independently at 12 months, crawled at 15 months, cruised at 18 months and walked at the age of 3 years. He has expressive language delay. At the age of 3 years and 6 months his comprehension was within normal limits, with no developmental concerns. He has moderate-severe sensorineural hearing loss, for which he has bilateral hearing aids. His primary dentition was late to erupt and an MRI of the facial bones performed at 3 years of age showed cysts in the lower jaw (Figure 2B). He had persistent pulmonary hypertension of the newborn, chronic lung disease and interstitial lung disease. He has a low CD8 T-lymphocyte count and receives prophylactic antibiotics during winters. He was found to have growth hormone deficiency and is on growth hormone supplementation (height and weight <0.4th centile at 3 ½ years). He has gastroesophageal reflux and a gastrostomy is being considered. He has a prominent forehead, broad nasal root, flat mid-face, thin upper lip, broad great toes, small nails and clinodactyly of 5th fingers. Trio WES performed using Agilent Sure Select Human All Exon Kit v6/Illumina NextSeq identified three *de novo* variants in the proband including a heterozygous exon 38 *KMT2D* c.1658G>T (p.(Gly3553Val)) variant. Two other *de novo* variants were identified both classed as likely benign (Table S2).

**Family 6:** Proband is a 6 year old male born to non-consanguineous parents (Figure 1) (Tables 1 and S1). He was born at 42 weeks gestation induced vaginal delivery and there were no neonatal problems. He was noted to have bilateral pre-auricular ear pits and small external ears with over-folded helices. He had bilateral clear response at his newborn hearing screen. Hearing assessments performed between the ages of 2½ and 4½ years revealed a mild bilateral progressive sensorineural hearing loss for which he was give bilateral hearing aids. Hypermetropia and left amblyopia, lacrimal drainage system dysgenesis, absent upper lid punctae, bilateral nasolacrimal sinuses and duct atresia, bifid uvula, mild hypothyroidism (with persistently raised TSH and normal free T4) were noticed. He also has a slight curvature of his fingers on both sides. A kidney ultrasound scan, renal function tests and an echocardiogram were normal.

His mother had a congenital abnormality of lacrimal ducts for which she required corrective surgery (Figure 1) (Table 1 and S1). She has bilateral sensorineural hearing loss. She has pre auricular pits and had surgery to have her external ears pinned back. She has a bifid uvula.

Trio WES Family 6 was performed as part of the Deciphering Developmental Disorders study^3^ (DDD #283413) and identified a heterozygous exon 39 *KMT2D* c.10745G>A (p.(Arg3582Gln)) variant in the proband and his mother.

**Family 7:** Proband is a 3 year old male born to non-consanguineous white British parents (Figures 1 and 2A) (Table 1 and S1). His mother’s pregnancy was complicated with colitis and pre-eclampsia. Intra-uterine growth retardation was noticed in the third trimester and the proband was born at 34 weeks gestation with weight of 1.8kgs (-1.31 SD). He had prolonged jaundice due to unconjugated hyperbilirubinemia and subsequent investigations revealed congenital hypothyroidism needing thyroxine replacement. Ultrasound scan of his neck showed normally located thyroid. He developed bilateral hydrocele that resolved without any intervention. He was found to have bilateral hypoplasia of lacrimal ducts in the neonatal period that needed correction by dacryocystorhinostomy. Branchial sinus was noticed soon after birth. The sinus leaked when he cried or sweated and required surgical correction. He has left sided moderate, likely mixed, hearing loss. He has a history of repeated ear and respiratory tract from a few months of age. He spoke his first words at the age of 14 months are was speaking in sentences from the age of 2½ years. Speech improved after grommets insertion and adenoidectomy. He had one episode of orbital cellulitis. He achieved head holding, smiling, motor milestones on time. A formal neuropsychological assessment identified cognitive, social, emotional and functional skills at an age-appropriate level of development. Specifically, his IQ was 104 and no KS1-like deficit in visuo-construction was identified. He has a small dimple just below the medial canthus of the left eye, laterally flared eyebrows, thin upper lip, and bilateral hypoplastic nipples. Targeted *KMT2D* sequencing was performed in the proband due to overlapping clinical features with other families identified in this study and a heterozygous exon 39 *KMT2D* c.10744C>T (p.(Arg3582Trp)) was detected which was confirmed by bi-directional Sanger sequencing. Parental testing showed that the variant had arisen *de novo*.

## Supplemental methods

**Peripheral blood DNA methylation:** All statistical analyses were performed in R 3.4.1 (www.r-project.org)^4^. Previously published studies on peripheral blood DNA methylation studies have been performed on Illumina Human Methylation 450K (hitherto 450K arrays)^5^. We removed all non-matching probes between the EPIC and 450K arrays using the Bioconductor package minfi^6^. Distribution of cell types within the whole blood samples were estimated and removed using the established Houseman method for each individual13. Initial data quality was measured by removing the methylated and unmethylated background signal levels exceeding the detection threshold of P > 0.01. In addition, we excluded cross-reactive probes^7^, probes on sex chromosomes and those that are age-associated^8-10.^ Raw beta values were logit transformed to M values following functional and quantile within array normalisations (SWAN)^11^. M values of all methylated positions were analysed using QLucore Omics Explorer 3.4 (QLucore, Lund, Sweden).

## Supplemental Figures

### Figure S1. CLUSTALW alignment of the central region of KMT2D

Alignment of amino acid residues 3477-3648 of human KMT2D (protein RefSeq NP_003473.3) against representative orthologues in vertebrate species, showing the high degree of phylogenetic conservation in the central region of KMT2D. RefSeq identifiers for all proteins are indicated on the left. Missense variants reported in this study for families 1 to 7 are indicted by red highlighting of residues, showing that these residues are highly conserved. Variant p.(Q3575H) was described in the separate study of Badalato et al. 2017^12^. A coiled-coil domain (residues 3562-3614) predicted by MARCOIL is indicated by yellow highlighting, and a coiled-coil dimer (residues 3506-3610) predicted by MultiCoil is indicated by underlined text. The region expressed in the recombinant KMT2D described in the text (residues 3503-3600) is highlighted by underlined text.

### Figure S2. Electropherograms for Family 4


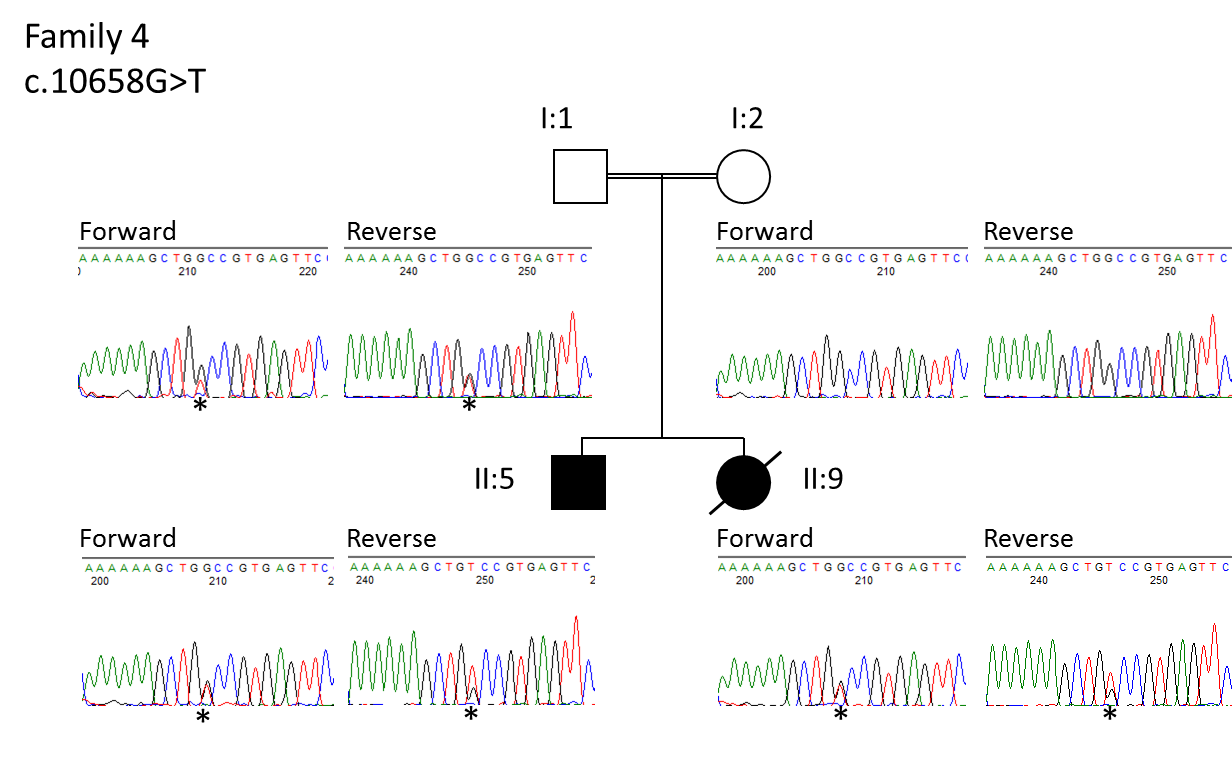


Sanger sequencing electropherograms showing the c.10658G>T variant in *KMT2D* in family members from Family 4. The position of the variant of interest is indicated with an asterisk. A heterozygous variant is present in the father and two affected siblings. Wildtype sequence is identified in the mother. Notice that the sequence peak height of the mutant (red ‘T’ peak) allele is reduced in father’s forward and reverse sequences when compared with the children’s sequence traces.

### Figure S3. DNA methylation comparison with Butcher et al., 2018


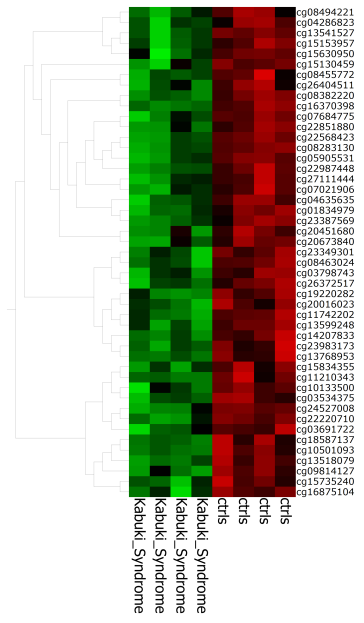

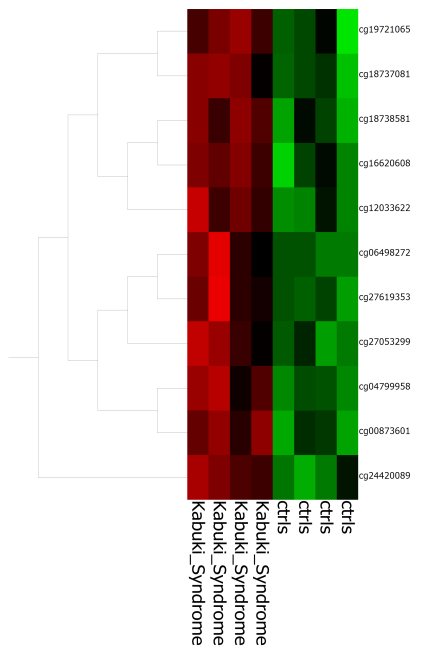


Kabuki syndrome

Controls

Kabuki syndrome

Controls

**B**

**A**

The hyper (A) and hypo (B) methylated CpG sites listed in Butcher et al., 2018 are detected to be similarly differentially methylated in our KS samples (green) in comparison with controls (blue) (p=0.001). In total we identified 421,891 (1,996 at 1x10^-9^<p<0.001) and 376,739 (2,489 at 1x10^-9^<p<0.001) DNA hypo- and hypermethylated positions (DMP) in the individuals with KS1 compared to controls.

### Figure S4. Missense *KMT2D* variants described in this study result in a DNA methylation signature distinct from type 1 Kabuki and CHARGE syndromes

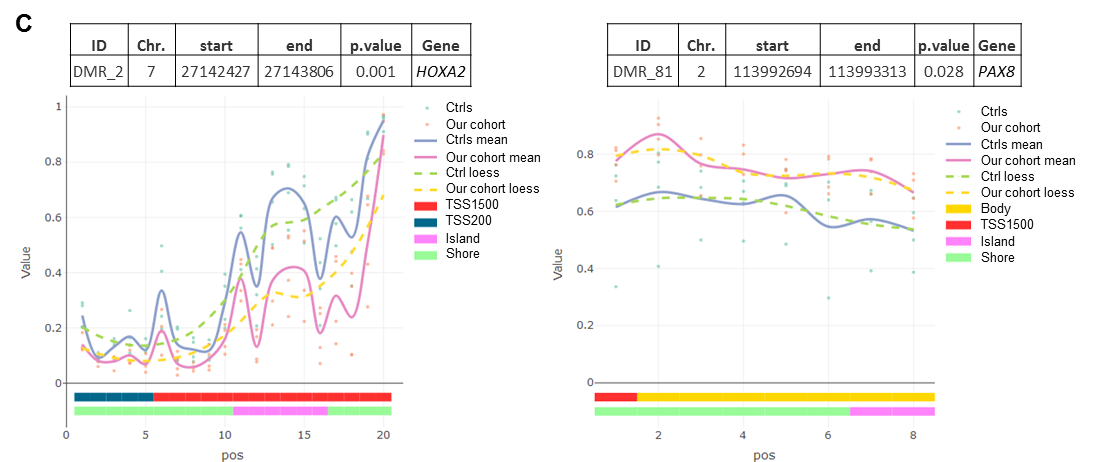


In total, we identified 8,679 (3,431 at 1x10^-9^<p<0.001) and 4,379 (1,731 at 1x10^-9^<p<0.001) DNA hypo- and hypermethylated positions in samples from the Ex38/39 KMT2D MV cohort against the controls.

A) Venn diagram shows significant difference between intersection analyses between hyper- and hypomethylated DMPs between Ex38/39 KMT2D MV cohort and type 1 Kabuki syndrome samples (p<0.001). B) Bar graph shows altered categories and functions of DMPs in our cohort compared to control samples. C) DMR plots, performed on using *ChAMP* package, show hypo and hypermethylated region corresponding to *HOXA2* and *PAX8,* respectively.

### Figure S5. Missense KMT2D variants described in this study do not result in decrease in KMT2D gene expression


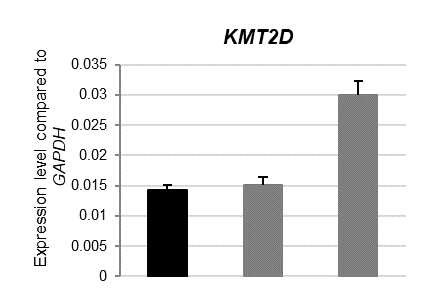


CF1 P2 P4

Quantitative real-time polymerase chain reaction (qRT-PCR) analysis of *KMT2D* transcript levels relative to *GAPDH* in fibroblast cells showed that the transcription levels in affected individuals’ cells are not decreased. RNA concentration was measured using a NanoDrop 2000 spectrophotometer (Thermo Scientific). 1 μg of RNA was reverse transcribed with random hexamer primers (Promega) to generate cDNA using the M-MLV Reverse Transcriptase kit (Promega), according to the manufacturer’s protocol. Quantitative real-time PCR (qRT-PCR) reactions were performed in triplicate on a Bio-Rad CFX394 Real Time system (Bio-Rad) using Power SYBR Green PCR Master mix (Applied Biosystems). For each sample, 2 µl cDNA (2 ng/µl) was incubated in a final volume of 10 µl with 5 µl of Power SYBR Green PCR Master mix (Applied Biosystems) and 0.5 µl of both forward and reverse target-specific primers (10 µM, Sigma). The expression of the target gene was evaluated using a relative quantification approach (2^-ΔC^_T_ method) with *GAPDH* as the internal reference for human genes.

### Figure S6. Missense KMT2D variants described in this study do not result in decrease in H3K4 tri-methylation levels


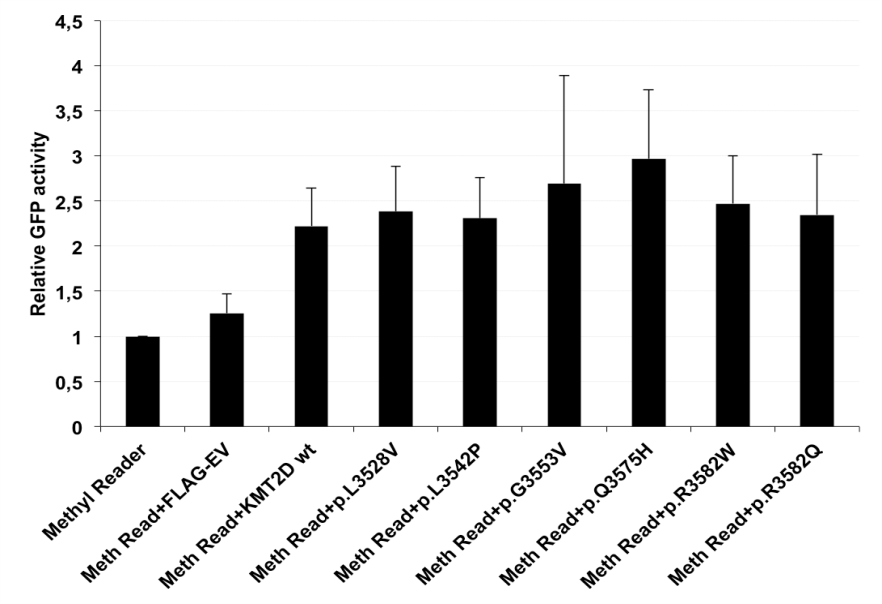


**B**

**A**


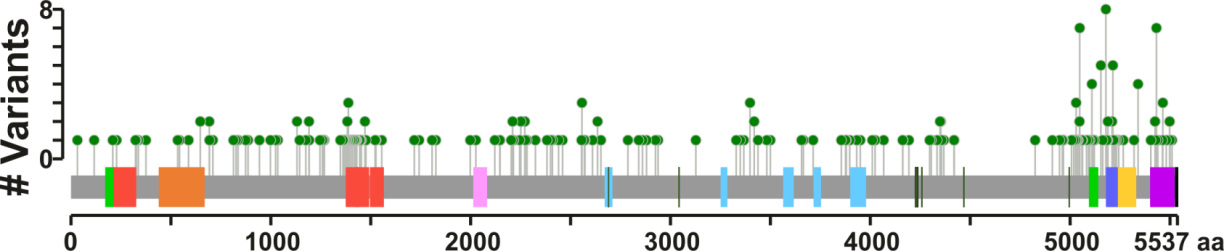


**Coiled coil**


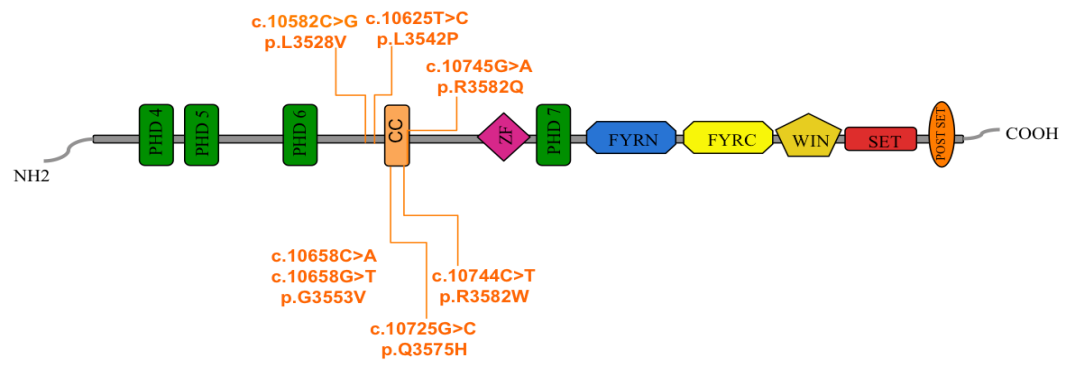


A) Distribution of the functionally analyzed *KMT2D* missense variants across the FUSION–KMT2D construct composed of PHD4–5–6 (amino acids 1358–1572), a new inserted region containing a CC domain (amino acids 3388–3697) and ZF-PHD7–FYRN–FYRC–WIN–SET–post-SET domains (amino acids 4507–5537). The inserted region is marked in orange. B) The H3K4me3 indicator show no variation in H3K4me3 activity for the 6 tested missense variants compared with the epigenetic reporter.

### Figure S7. Protein secondary structure of KMT2D wild-type protein


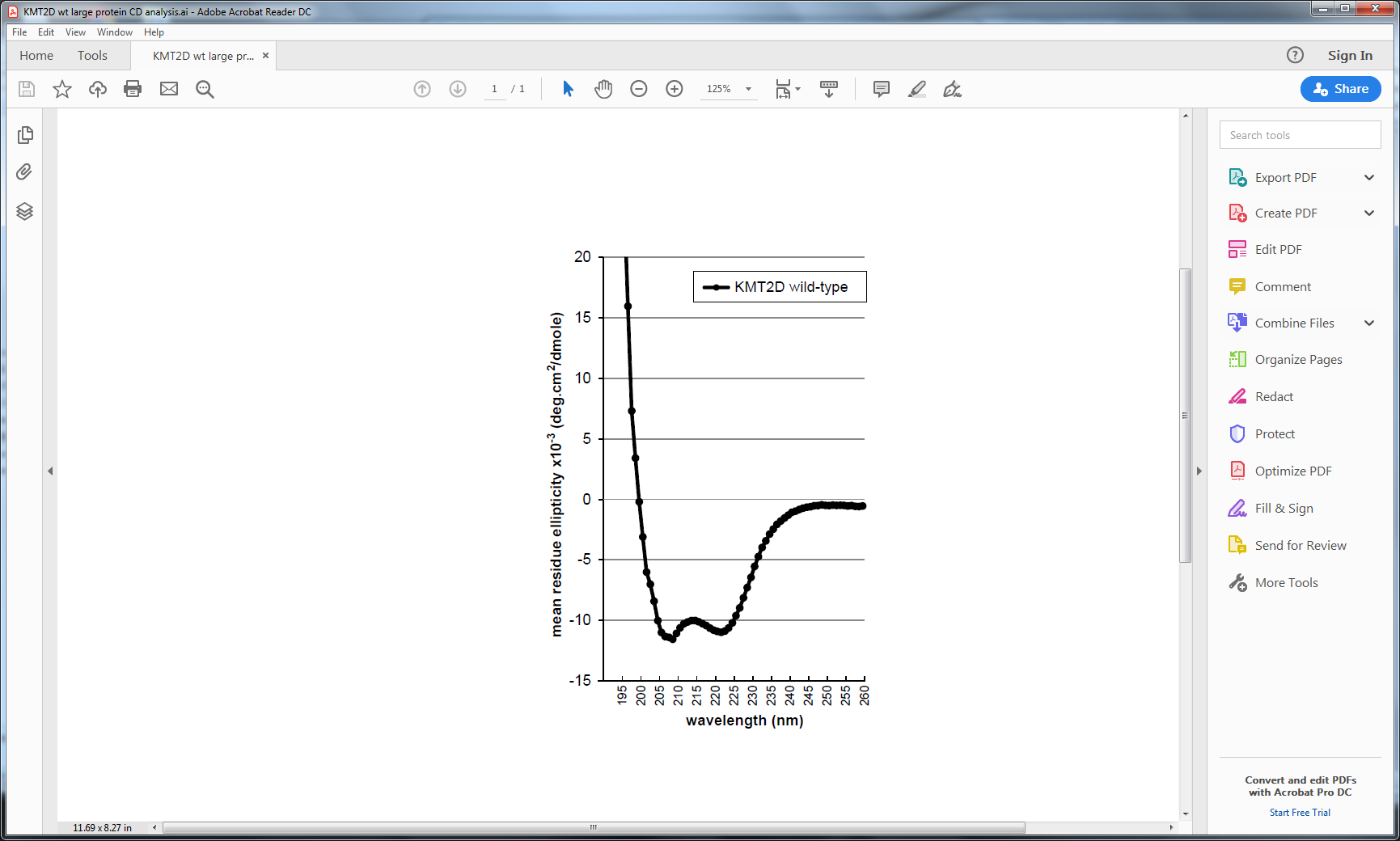


CD spectroscopy trace of recombinant KMT2D wild-type protein fragment (residues 3231-3600, molecular weight 40.7kDa) with the following proportions of predicted secondary structure: 0.22 alpha helix, 0.18 beta strand, 0.19 turn and 0.41 disordered.

## Supplementary Tables

## Table S1. Clinical features of individuals with missense *KMT2D* variants

| **Demographics** | | | **Genotype information** | | **Cranio-facial and dental** | | | | | | | |
| --- | --- | --- | --- | --- | --- | --- | --- | --- | --- | --- | --- | --- |
| **Fam#** | **Sex** | **Age at last assessment** | **gDNA (hg19); *KMT2D* cDNA; Exon number; KMT2D protein** | **Inheritance** | **Branchial** | **Oto** | **Ocular** | **Lacrimo** | **Naso** | **Palate** | **Teeth** | **Dysmorphism** |
| 1 | F | 13 yr | 12:49428008G>C; c.10582 C>G; ex 38; p.(Leu3528Val) | de novo | Bilateral branchial arch fistulas. | Severe sensoineural hearing loss. Low-set ears with underdeveloped and overfolded helix. Ear pits. | Right incomplete iridal coloboma | Unilateral narrow naso-lacrimal duct | Bilateral choanal atresia |  |  | Mild dolichocephaly, tall forehead, sparse eyebrows, down-slanted palpebral fissures, depressed nasal bridge, low hanging columella, broad philtrum, low-set ears, thin upper lip, moderate micrognathia. Bilateral single palmar creases |
| 2 | M | 2y8m | 12:49428008G>C; c.10582C>G; ex 38; p.(Leu3528Val) | de novo | Bilateral microtia with sinuses discharing intermittently. Simuses situatioed between the two rudimentary bars representing his pinnae at the level of the tragus but posterior to it. | Bilateral grade 3 microtia with sinuses discharing intermittently. Bilateral mod mixed sensorineural and conductive hearing loss. MRI bilateral post semi-circular canal hypoplasia with normal cochlea and vestibule. | No abnormality | Obstructed lacrimal duct on left, thought to be secondary to broadened nasal bridge and choanal atresia | Left choanal atresia dilated multiple times. Antrior rhinoscopy reveals bilateral nasal misting. |  | 8 rotten primary dentition removed | Left LMN facial palsy (hypoplastic facial nerve). Micrognathia |
| 3 | M | 28 days | 12:49427965A>G; c.10625T>C; ex 38; p.(Leu3542Pro) | de novo | A sinus coming out the side of his face | Bilateral microtia with normal inner ear canals | Microphthalmia/colobomata and hardly any eye tissue |  | Choanal stenosis | No abnormality | RIP prior to dentition | Extreme micrognathia |
| 4 | M | 9y | 12:49427932C>A; c.10658G>T; ex 38; p. (Gly3553Val) | Paternal | Neck pits | Recurrent otitis media, bilateral conductive hearing loss. | No abnormality | Absent upper lacrimal duct with bilateral duct stenosis. | Unilateral right choanal atresia | no abnormality | Overcrowded teeth. | Sparse hair and eyebrows, downslanting palpebral fissures, low-set ears, hypo-plastic alai nasi. Long eyelashes. Dystrophic nails. |
| 4 | F | 4m | 12:49427932C>A; c.10658G>T; ex 38; p. (Gly3553Val) | Paternal | Neck pits | Hearing loss | No abnormality | Absent lacrimal duct | Bilateral choanal atresia | no abnormality | No abnormality | Widely spaced eyes, depressed nasal bridge, low set right ear, sparse hair and eyebrows. Long eyelashes. |
| 4 | M | 39y | 12:49427932C>A; c.10658G>T; Mosaic 38; p. (Gly3553Val) | Unknown | No abnormality | No abnormality | No abnormality | No abnormality | No abnormality | No abnormality | No abnormality | No abnormality |
| 5 | M | 3.5y | 12:49427932C>A; c.10658G>T; ex 38; p. (Gly3553Val) | de novo | Branchial cysts | Pre-auricular pits, moderate-severe hearing loss; grommets insertion. | No abnormality | No abnormality | Bilateral choanal atresia | no abnormality | Neonatal teeth. Delayed dentition. Cysts lower jaw. | Low set ears, thin upper lip. |
| 6 | M | 6y | 12:49427743C>T; c.10745G​>A; ex 38; p.(Arg3582Gln) | Maternal | No abnormality | Mild bilateral sensorineural hearing loss, small external ears with overfolded helices and ear pits. | Hypermetropia and left amblyopia | Lacrimal drainage system dysgenesis, absent upper lid punctae, bilateral nasolacrimal sinuses |  | Bifid uvula |  |  |
| 6 | F | N/A | 12:49427743C>T; c.10745G​>A; ex 38; p.(Arg3582Gln) | Maternal | No abnormality | Mild bilateral sensorineural hearing loss, small external ears with overfolded helices and ear pits. |  | Lacrimal drainage system dysgenesis, absent upper lid punctae, bilateral nasolacrimal sinuses |  | bifid Uvula |  |  |
| 7 | M | 3y 5m | 12:49427744G>A; c.10744C>T; ex 38; p.(Arg3582Trp) | de novo | Branchial sinus | Conductive hearing loss and normal external ears | No abnormality | Bilateral lacrimal duct atresia | Bilateral choanal atresia | Normal | No abnormality | A small dimple just below the medial canthus of the left eye, prominent occipit, laterally flared eyebrows, thin upper lip |

|  | **Physical anomalies** | | | | | **Neuro-developmental** | | | | **Feeding and growth** | | | | | **Other systems and comments** | | |  |  |
| --- | --- | --- | --- | --- | --- | --- | --- | --- | --- | --- | --- | --- | --- | --- | --- | --- | --- | --- | --- |
| **Fam#** | **Mammary** | **Heart** | **Kidneys** | **Genitalia** | **GI** | **CNS** | **Motor delay** | **Speech delay** | **Intellectual disability** | **Feeding difficulties** | **FTT** | **Height cm (centile, age)** | **Weight kg (centile, age)** | **OFC cm (centile, age)** | **Endocrine** | **Immunity** | **Other comments** | **Gnomad** | **COSMIC** |
| 1 | Bilateral athelia | No abnormality | Normal ultrasound | No abnormality | No abnormality |  | no | yes | no | No abnormality |  | 10 years height <10th percentile | N/A | N/A | Hypothyroidism. Growth hormone replacement although no evidence of GH deficiency. |  | Moderate thoracic scoliosis | not present | not present |
| 2 | Missing nipple on left | No abnormality | Normal renal USS | No abnormality | Gastro-oeso reflux when younger | L LMN facial palsy | no | yes | no | Weak suck as a baby and had NG tube temporarily. |  | 55cm (2nd centile) at 10 weeks | BW < 0.4th centile. 10.15kg at 18m (25th centile) | 38.6 cm (2nd centile) at 10 weeks |  | More coughs and colds than siblings but no major infections | Mild external deviation of terminal phalange of thumb | not present | not present |
| 3 | No abnormality | Patent ductus and pulmonary hypertension but structurally normal heart | Normal ultrasound | No abnormality | No abnormality | Brain MRI normal parenchyma | RIP prior to being able to assess | RIP prior to being able to assess | RIP prior to being able to assess | Could not feed orally | RIP prior to being able to assess | N/A | 2.62kg at birth | 37 cms at birth | Small ant pituitary | RIP prior to being able to assess |  | not present | not present |
| 4 | Hypoplastic nipples, absent breast tissue. | Small PFO, right ventricular hypertrophy | Normal renal USS | Left undescended testis. | Chronic diarrhoea | Normal cranial CT | not known | Speech delay | no abnormality | No abnormality | Yes | <5th centile | <5th centile | N/A | Small thyroid gland. TFTs normal. | chronic infections, recurrent chest infections. | Skeletal survey; prominent frontoparietal region. | not present | not present |
| 4 | Hypoplastic nipples, absent breast tissue. | PPHN | Normal renal USS | No abnormality | No abnormality | Normal cranial CT | N/A | N/A | N/A | No abnormality | Yes | 52 (3 mths, < 3rd centile) | 2.3 (At 3 mnth, <3rd centile) | N/A | Absent thyroid | Chronic infections | BW 2.32kg at 37/40 (<10th centile). Died at 4 months of age following pneumonia. | not present | not present |
| 4 | No abnormality | No abnormality | No abnormality | No abnormality | No abnormality | No abnormality | not known | not known | No abnormality | No abnormality | No | N/A | N/A | N/A | No abnormality | No abnormality |  | not present | not present |
| 5 | Athelia | ASD, PPHN | No abnormality | no abnormality | Absent gall bladder. Dilated bile duct. | Caudothalamic cyst | yes, sat at 12m, crawled 15m, cruising 18m, walked at 3 yrs. | Delayed expressive language | No | Poor growth and feeding, awaiting gastrostomy placement | Yes | 0.4th centile at 4 yrs, having GH tx. | <0.4th centile at 4 yrs | 50th centile at 4 yrs | Congenital Hypothyroidism | Absent thymus, low CD8 cell count. | Echogenic bowel. Induced at 37/40, concerns about fetal blood flow. BW 2.35kg. Recurrent breath-holding attacks at age 4yrs. | not present | not present |
| 6 |  | No abnormality | Normal ultrasound |  |  |  |  |  |  |  |  | N/A | N/A | N/A | Hypothyroidism |  | Clinical suspicion of BOR syndrome. | not present | COSM4604032, c.10745G>A, p.R3582Q (oesophagus). |
| 6 |  | No abnormality | Normal ultrasound |  |  |  |  |  |  |  |  | N/A | N/A | N/A |  |  | Clinical suspicion of BOR syndrome | not present | COSM4604032, c.10745G>A, p.R3582Q (oesophagus) |
| 7 | Bilateral hypoplastic nipples |  | Normal | Bilateral hydrocele which resolved without any treatment |  |  | No | No | No | GOR | No | 93 (9th - 25th cent; 3y 5m) | 13.6 (25th; 3y 5m) | 50.5 (9th - 25th; 3y 5m) | Congenital hypothyroidism | Recurrent ENT infections | Ex preterm 34 weeks. prolonged jaundice due to unconjugated. Clinical suspicion of BOR syndrome. hyperbilirubinemia | not present | COSM431206, C.10744C>T, P.R3582W (breast) |

(N/A: not applicable)

### Table S2. *De novo* and rare biallelic variants from the exome/genome sequencing from families 2-5

| **Fam** | **Variant Type** | **Gene** | **Nomenclature** | **Protein Nomenclature** | **rs number** |
| --- | --- | --- | --- | --- | --- |
| 2 | X-linked | *THOC2* | X:122778483T>C, NM_001081550.1:c.1604A>G | NP_001075019.1:p.His535Arg | rs750213064 |
|  | compound heterozygous | *TTN* | 2:179425894C>T, NM_133378.4:c.77261G>A, NM_133437.4:c.58346G>A, NM_133437.3:c.58346G>A, NM_003319.4:c.57770G>A, NM_133432.3:c.58145G>A, NM_001267550.2:c.84965G>A, NM_001267550.1:c.84965G>A, NM_001256850.1:c.80042G>A | NP_596869.4:p.Arg25754His, NP_597681.4:p.Arg19449His, NP_003310.4:p.Arg19257His, NP_597676.3:p.Arg19382His, NP_001254479.2:p.Arg28322His, NP_001243779.1 | rs373532064 |
|  | compound heterozygous | *TTN* | 2:179391992A>G, NM_133378.4:c.100019T>C, NM_133437.4:c.81104T>C, NM_133437.3:c.81104T>C, NM_003319.4:c.80528T>C, NM_133432.3:c.80903T>C, NM_001267550.2:c.107723T>C, NM_001267550.1:c.107723T>C, NM_001256850.1:c.102800T>C | NP_596869.4:p.Ile33340Thr, NP_597681.4:p.Ile27035Thr, NP_003310.4:p.Ile26843Thr, NP_597676.3:p.Ile26968Thr, NP_001254479.2:p.Ile35908Thr, NP_001243779.1:p.Ile34267Thr | rs769141222 |
|  | homozygous | *HLA-DRB5* | 6:32489744G>C, NM_002125.3:c.308C>T, NM_002125.3:c.308C>G, NM_002125.3:c.308C>A, NM_022555.3:c.308G>C, NM_022555.3:c.308G>T, NM_022555.3:c.308G>A, | NP_002116.2:p.Ala103Val, NP_002116.2:p.Ala103Gly, NP_002116.2:p.Ala103Glu | rs1059598 |
|  | X-linked | *FMR1* | X:147019111G>A, NM_002024.5:c.1117G>A, NM_001185076.1:c.1117G>A, NM_001185082.1:c.1117G>A | NP_002015.1:p.Val373Ile, NP_001172005.1:p.Val373Ile, NP_001172011.1:p.Val373Ile, NP_001172004.1:p.Val373Ile, NP_001172010.1:p.Val373Ile | rs782257390 |
|  | homozygous | *APAF1* | 12:99093210G>A, NM_013229.2:c.2296G>A, NM_001160.2:c.2296G>A, NM_181861.1:c.2329G>A, NM_181868.1:c.2329G>A | NP_037361.1:p.Glu766Lys, NP_001151.1:p.Glu766Lys, NP_863651.1:p.Glu777Lys, NP_863658.1:p.Glu777Lys | rs138526583 |
|  | X-linked | *NHS* | X:17653697C>T, NM_001136024.3:c.11C>T, NM_001136024.2:c.11C>T, NM_001291868.1:c.11C>T | NP_001129496.1:p.Ala4Val, NP_001278797.1:p.Ala4Val | rs143065064 |
| 3 | homozygous | *RNF212* | NM_001193318.2:c.721_722insGGGCAG, NM_001193318.2:c.721_722insGGCTGGCTCTAGCCTGGGCAG, NM_001193318.2:c.721_722insGGCTCCAGCCTGGGCAG, NM_001193318.2:c.721_722insGGTTGGCTCCAGCCTGGGCAG, NM_001193318.2:c.721_722insGGCTGGCTCCAGCCTGGGCAG, NM_001193318.2:c.721_722insGGCTGGCTCCAGCCAGGGCAG | NP_001180247.1:p.Ser241delinsTrpAlaAla, NP_001180247.1:p.Ser241delinsTrpLeuAlaLeuAlaTrpAlaAla, NP_001180247.1:p.Ser241fs, NP_001180247.1:p.Ser241delinsTrpLeuAlaProAlaTrpAlaAla, NP_001180247.1:p.Ser241delinsTrpLeuAlaProAlaTrpAlaAla, NP_001180247.1:p.Ser241delinsTrpLeuAlaProAlaArgAlaAla | rs138488801 |
|  | De novo | *FCN1* | NM_002003.4; c.732G>A | NP_001994.2 :p.= |  |
|  | De novo | *CACFD1* | NM_001135775.2:c.39C>G NM_001242369.1:c.39C>G NM_001242370.1:c.39C>G NM_017586.3:c.39C>G | NP_001129247.1 :p.Ser13Arg NP_001229298.1:p.Ser13Arg NP_001229299.1:p.Ser13Arg NP_060056.1:p.Ser13Arg |  |
|  | De novo | *MAVS* | NM_020746.4:c.1064G>A, NM_020746.4:c.1064G>T, NR_037921.1:n.1063G>A, NR_037921.1:n.1063G>T, NM_001206491.1:c.641G>A, NM_001206491.1:c.641G>T | NP_065797.2:p.Arg355His, NP_065797.2:p.Arg355Leu, NP_001193420.1:p.Arg214His, NP_001193420.1:p.Arg214Leu | rs143802036 |
| 4 | homozygous | *CCDC114* | NM_144577.3; c.586C>T | NP_653178.3:p.Arg196Trp | rs200168343 |
|  | homozygous | *C3orf33* | NM_173657.2:c.307C>T, NM_173657.1:c.307C>T | NP_775928.1:p.Leu103Phe | rs374827619 |
|  | homozygous | *KLHL24* | NM_001349418.1:c.704G>A, NM_001349428.1:c.-263G>A, NM_001349417.1:c.704G>A, NM_001349429.1:c.-263G>A, NM_001349413.1:c.704G>A, NM_001349414.1:c.704G>A, NM_001349420.1:c.704G>A, NM_001349416.1:c.704G>A, NM_001349424.1:c.704G>A, NM_001349415.1:c.704G>A, NM_001349422.1:c.704G>A, NM_001349425.1:c.704G>A, NM_001349423.1:c.704G>A, NM_001349421.1:c.704G>A, NM_001349426.1:c.704G>A | NP_001336348.1:p.Arg235His, NP_001336347.1:p.Arg235His, NP_001336346.1:p.Arg235His, NP_001336342.1:p.Arg235His, NP_001336343.1:p.Arg235His, NP_001336349.1:p.Arg235His, NP_001336345.1:p.Arg235His, NP_001336353.1:p.Arg235His, NP_001336344.1:p.Arg235His, NP_001336351.1:p.Arg235His, NP_001336354.1:p.Arg235His, NP_001336352.1:p.Arg235His, NP_001336350.1:p.Arg235His, NP_001336355.1:p.Arg235His | rs372055673 |
|  | homozygous | *ANKFN1* | NM_001365758.1; c.2606C>A | NP_001352687.1; p.P869Q |  |
|  | homozygous | *COL6A5* | NM_153264.6:c.481G>A, NM_001278298.1:c.481G>A | NP_694996.5:p.Gly161Arg, NP_001265227.1:p.Gly161Arg | rs777760382 |
| 5 | de novo | *RBM25* | 14:7354417C>T, NM_021239.3; c.254C>T | NP_067062; p.P85L |  |
|  |  | *PTPRM* | 18:8384644A>G; NM_001105244.1; c.4004A>G | NP_002836; p.D1322G |  |
| 6 | de novo | *PTPN22* | 1:114394681G>A; NM_015967.6:c.796C>T, NM_015967.6:c.796C>A, NM_015967.5:c.796C>T, NM_015967.5:c.796C>A, NM_001193431.2:c.796C>T, NM_001193431.2:c.796C>A, NM_001193431.1:c.796C>T, NM_001193431.1:c.796C>A, NM_001308297.1:c.724C>T, NM_001308297.1:c.724C>A | NP_057051.3:p.Arg266Trp, NP_001180360.1:p.Arg266Trp, NP_001295226.1:p.Arg242Trp | rs72650670 |
|  |  | *YTHDF1* | 20:61834909G>A; NM_017798.3:c.383C>T | NP_060268.2:p.Ala128Val | rs146457378 |
|  | compound heterozygous | *ZNF724* | 19:23405270T>C, NM_001355406.1:c.1759A>G, NM_001355404.1:c.1777A>G, NM_001355405.1:c.1585A>G | NP_001342335.1:p.Lys587Glu, NP_001342333.1:p.Lys593Glu, NP_001342334.1:p.Lys529Glu, | rs758059045 |
|  |  | *ZNF724* | 19:23405893T>C, NM_001355406.1:c.1136A>G, NM_001355406.1:c.1136A>C, NM_001355404.1:c.1154A>G, NM_001355404.1:c.1154A>C, NM_001355405.1:c.962A>G, NM_001355405.1:c.962A>C | NP_001342335.1:p.Lys379Arg, NP_001342335.1:p.Lys379Thr, NP_001342333.1:p.Lys385Arg, NP_001342333.1:p.Lys385Thr, NP_001342334.1:p.Lys321Arg, NP_001342334.1:p.Lys321Thr, | rs773214910 |
|  | maternally inherited | *WLS* | 1:68611633C>T: NM_001002292.3; c.1438G>A NM_001193334.1; c.1171G>A NM_024911.6; c.1499G>A | NP_001002292.3:p.M397I, NP_001180263.1:p.M308I, NP_079187.3:p.M399I |  |
|  |  | *FAM78B* | 1:166135293T>A: NM_001017961.4:c.193A>T, NM_001017961.3:c.193A>T | NP_001307231.1:p.Thr65Ser | rs1172428923 |
|  |  | *DISP1* | 1:223176145T>C: NM_032890.4:c.1406T>C, NM_032890.3:c.1406T>C, NM_001350630.1:c.677T>C | NP_116279.2:p.Leu469Ser, NP_001337559.1:p.Leu226Ser | rs200592414 |
|  |  | *MARS2* | 2:198570280ATTTCT>A; NM_138395.3:c.254_259ATTTCT>A | NP_612404.1:p.I51_Y53delinsN |  |
|  |  | *MARS2* | 2:198570287AC>A; NM_138395.3:c.261AC>A | NP_612404.1:pY53fs |  |
|  |  | *MARS2* | 2:198570290TG>T; NM_138395.3:c.264TG>T | NP_612404.1:pV54fs |  |
|  |  | *MARS2* | 2:198570292AACGCGG>A; NM_138395.3:c.266_271AACGCGG>A | NP_612404.1:p.N55_A57delinsT |  |
|  |  | *CPS1* | 2:211467041A>G: NM_001875.4:c.1823A>G, NM_001122634.3:c.470A>G, NM_001122634.2:c.470A>G, NM_001122633.2:c.1841A>G | NP_001866.2:p.Asp608Gly, NP_001116106.1:p.Asp157Gly, NP_001116105.1:p.Asp614Gly | rs369748670 |
|  |  | *WDR19* | 4:39226654G>A: NM_001317924.1G>A NM_025132.3G>A |  |  |
|  |  | *FILIP1* | 6:76124437G>GAGTA: NM_015687.4:c.248_251dup, NM_015687.2:c.248_251dup, NM_001300866.2:c.248_251dup, NM_001289987.2:c.257_260dup | NP_056502.1:p.Ser85fs, NP_001287795.1:p.Ser85fs, NP_001276916.1:p.Ser88fs | rs752141570 |
|  |  | *HGF* | 7:81335690C>T: NM_000601.4:c.1835G>A NM_000601.5:c.1897G>A NM_001010932.1:c.1882G>A NM_001010932.2:c.1822G>A | NP_000592.3:p.G557E, NP_001010932.1:p.G552E |  |
|  |  | *CYP3A4* | 7:99367466A>C; NM_001202855.2:p.553T>G NM_017460.5:p.553T>G | NP_001189784.1 :p.I553S, NP_059488.2:p.I553S |  |
|  |  | *ST18* | 8: 53071579C>A; NM_014682.2:c.2505G>T | NP_055497.1:p.S562I |  |
|  |  | *ST18* | 8: 53071618C>A: NM_014682.2:c.2466G>T | NP_055497.1:p.G549V |  |
|  |  | *KIAA2026* | 9: 5920329C>G:  NM_001017969.2:c.5667G>T, NM_001017969.2:c.5667G>C | NP_001017969.2:p.Gln1889His, NP_001017969.2:p.Gln1889His | rs371270457 |
|  |  | *CEP78* | 9: 80858454C>T: NM_001330691.2:c.680C>T, NM_001330693.2:c.680C>T, NM_001098802.2:c.680C>T, NM_001098802.1:c.680C>T, NM_032171.2:c.680C>T, NM_032171.1:c.680C>T, NM_001349839.1:c.680C>T, NM_001349840.1:c.680C>T, NM_001349838.1:c.680C>T, NM_001330694.1:c.680C>T | NP_001317620.1:p.Ala227Val, NP_001317622.1:p.Ala227Val, NP_001092272.1:p.Ala227Val, NP_115547.1:p.Ala227Val, NP_001336768.1:p.Ala227Val, NP_001336769.1:p.Ala227Val, NP_001336767.1:p.Ala227Val, NP_001317623.1:p.Ala227Val | rs368402315 |
|  |  | *LGALS12* | 11: 63283178T>C: NM_033101.3:c.857T>A, NM_033101.3:c.857T>C, NM_001142537.1:c.674T>A, NM_001142537.1:c.674T>C, NM_001142538.1:c.647T>A, NM_001142538.1:c.647T>C, NM_001142535.1:c.860T>A, NM_001142535.1:c.860T>C, NM_001142536.1:c.830T>A, NM_001142536.1:c.830T>C | NP_149092.2:p.Phe286Tyr, NP_149092.2:p.Phe286Ser, NP_001136009.1:p.Phe225Tyr, NP_001136009.1:p.Phe225Ser, NP_001136010.1:p.Phe216Tyr, NP_001136010.1:p.Phe216Ser, NP_001136007.1:p.Phe287Tyr, NP_001136007.1:p.Phe287Ser, NP_001136008.1:p.Phe277Tyr, NP_001136008.1:p.Phe277Ser | rs146548773 |
|  |  | *CEP290* | 12: 88520200T>C; NM_025114.3:c.1302A>G | NP_079390.3:p.K320E |  |
|  |  | *JPH4* | 14: 24040357C>A; NM_001146028.1:c.2375G>T | NP_001139500.1:p.G528V |  |
|  |  | *SETD1A* | 16: 30975995G>A: NM_014712.2:c.932G>A, NM_014712.1:c.932G>A | NP_055527.1:p.Arg311His | rs764044469 |
|  |  | *C16orf58* | 16: 31503334C>G: NM_022744.3:c.1351G>C | NP_073581.2:p.L435F |  |
|  |  | *ELMO3* | 16: 67237746A>G: NM_024712.3:c.2288A>G | NP_078988.2:p.Asn763Ser | rs374788979 |
|  |  | *MPO* | 17:56357813C>CA; NM_000250.1:c.339G>TG | NP_000241.1 :p.L54LX |  |
|  |  | *ATP13A1* | 19: 19767949C>A: NM_020410.2:c.760G>T | NP_065143.2:p.Val254Leu | rs1459808873 |
|  |  | *URI1* | 19: 30433504C>T: NM_003796.3:c.50C>T | NP_003787.2:p.Ala17Val | rs773692912 |
|  |  | *ZNF865* | 19: 56125579G>A: NM_001195605.1:c.595G>A | NP_001182534.1:p.Ala199Thr | rs1013767552 |
|  |  | *SOGA1* | 20: 35438434C>T: NM_080627.3:c.2534G>T, NM_080627.3:c.2534G>A, NM_080627.2:c.2534G>T, NM_080627.2:c.2534G>A, NM_199181.2:c.1820G>T, NM_199181.2:c.1820G>A | NP_542194.2:p.Arg845Leu, NP_542194.2:p.Arg845Gln, NP_954650.2:p.Arg607Leu, NP_954650.2:p.Arg607Gln | rs370773329 |
|  |  | *ITGB2* | 21: 46330318G>A: NM_001303238.1:c.-180C>T |  | rs377614767 |

### Table S3. List of samples used for DNA methylation studies and proliferation and migration assays

| **DNA methylation array – blood samples** | | | | | | | |
| --- | --- | --- | --- | --- | --- | --- | --- |
| **Sample n** | **Sample Group** | **Sex** | | **age** | **Mutation** | | **Family n** |
| 1 | controls | F | | 5y |  | |  |
| 2 | controls | F | | 22y |  | |  |
| 3 | controls | F | | 14y |  | |  |
| 4 | controls | M | | N/A |  | |  |
| 5 | CHARGE syndrome | F | | 4days | CHD7 nonsense mutation | |  |
| 6 | CHARGE syndrome | F | | 22y | CHD7 c.282delT, p.(Asn96Thrfs*115) | |  |
| 7 | CHARGE syndrome | M | | 11y | CHD7 c.5050G>A slice site mutation | |  |
| 8 | CHARGE syndrome | F | | 6days | CHD7 c.7957C>T p (A r9 2653Ter) | |  |
| 9 | Our cohort | M | | 3y | c.10658C>A p.(Gly3553Val) | | 5 |
| 10 | Our cohort | M | | 4y | c.10744C>T p.(Arg3582Trp) | | 7 |
| 11 | Our cohort | M | | 6y | c.10745G​>A p.(Arg3582Gln) | | 6 |
| 12 | Our cohort | M | | 1y | c.10625T>C p.(Leu3542Pro) | | 3 |
| 13 | Kabuki Syndrome | F | | 4 months | KMT2D het c.8727_8730AAGT | |  |
| 14 | Kabuki Syndrome | F | | 6y | KMT2D het c.9581delA | |  |
| 15 | Kabuki Syndrome | M | | 6y | KMT2D het c.12179_12182delCTGA | |  |
| 16 | Kabuki Syndrome | M | | 10y | KMT2D het c.16019G>A; p.(Arg5340Gln) | |  |
|  | | | | | | | |
| **Proliferation and migration assay – fibroblast samples** | | | | | | | |
| **Sample name** | **Sample Group** | | **Mutation** | | | **Family n** | |
| CF1 | Control fibroblast | |  | | |  | |
| P2 | NewKMT2D_Syndrome | | c.10744C>T p.(Arg3582Trp) | | | 7 | |
| P4 | NewKMT2D_Syndrome | | c.10625T>C p.(Leu3542Pro) | | | 3 | |

### Table S4. List of DMR in our cohort of affected individuals with missense *KMT2D* variants

| **ID** | **Chr** | **start** | **end** | **width** | **area** | **L** | **p.value** | **p.valueArea** |
| --- | --- | --- | --- | --- | --- | --- | --- | --- |
| DMR_1 | 17 | 7832680 | 7833663 | 983 | 22.1076 | 9 | 0.00067 | 0.00328 |
| DMR_2 | 7 | 2.7E+07 | 2.7E+07 | 1379 | 27.8243 | 20 | 0.00076 | 0.00198 |
| DMR_3 | 4 | 1.6E+08 | 1.6E+08 | 824 | 18.3275 | 8 | 0.00103 | 0.00534 |
| DMR_4 | 1 | 2.3E+08 | 2.3E+08 | 717 | 15.3285 | 7 | 0.00128 | 0.0088 |
| DMR_5 | 6 | 3E+07 | 3E+07 | 610 | 22.2434 | 23 | 0.00102 | 0.00324 |
| DMR_6 | 17 | 7.8E+07 | 7.8E+07 | 505 | 14.7101 | 7 | 0.00146 | 0.00991 |
| DMR_7 | 11 | 2292751 | 2293552 | 801 | 19.2777 | 17 | 0.00138 | 0.00468 |
| DMR_9 | 6 | 3.3E+07 | 3.3E+07 | 584 | 16.7529 | 15 | 0.00202 | 0.00671 |
| DMR_10 | 19 | 5.2E+07 | 5.2E+07 | 979 | 16.2304 | 14 | 0.00257 | 0.00744 |
| DMR_11 | 1 | 3.6E+07 | 3.6E+07 | 1417 | 16.2015 | 14 | 0.00259 | 0.00744 |
| DMR_12 | 16 | 8806359 | 8807308 | 949 | 15.8818 | 15 | 0.0023 | 0.00799 |
| DMR_13 | 19 | 4.7E+07 | 4.7E+07 | 796 | 13.5087 | 7 | 0.00221 | 0.01274 |
| DMR_14 | 22 | 5.1E+07 | 5.1E+07 | 1046 | 14.8411 | 15 | 0.00272 | 0.00964 |
| DMR_16 | 17 | 3.4E+07 | 3.4E+07 | 1015 | 20.0533 | 13 | 0.00189 | 0.00423 |
| DMR_18 | 10 | 1E+08 | 1E+08 | 624 | 18.2412 | 13 | 0.00241 | 0.00538 |
| DMR_19 | 6 | 1.1E+07 | 1.1E+07 | 841 | 15.2431 | 9 | 0.0031 | 0.00895 |
| DMR_20 | 22 | 4.6E+07 | 4.6E+07 | 821 | 17.043 | 12 | 0.00323 | 0.0065 |
| DMR_21 | 19 | 3.8E+07 | 3.8E+07 | 265 | 12.62 | 8 | 0.00463 | 0.01558 |
| DMR_22 | 6 | 3.2E+07 | 3.2E+07 | 253 | 16.3737 | 12 | 0.00359 | 0.00724 |
| DMR_23 | 17 | 5.8E+07 | 5.8E+07 | 232 | 10.8477 | 7 | 0.00596 | 0.02379 |
| DMR_24 | 3 | 5.2E+07 | 5.2E+07 | 732 | 12.1219 | 8 | 0.00554 | 0.01755 |
| DMR_25 | 1 | 5.1E+07 | 5.1E+07 | 601 | 12.9466 | 9 | 0.00597 | 0.01451 |
| DMR_26 | 6 | 3.1E+07 | 3.1E+07 | 285 | 11.5173 | 8 | 0.00715 | 0.02007 |
| DMR_27 | 12 | 1.1E+08 | 1.1E+08 | 612 | 14.2885 | 10 | 0.00482 | 0.01072 |
| DMR_28 | 3 | 1.6E+08 | 1.6E+08 | 1047 | 14.1236 | 10 | 0.00499 | 0.01106 |
| DMR_29 | 17 | 170770 | 171257 | 487 | 12.4685 | 9 | 0.00673 | 0.01613 |
| DMR_30 | 7 | 2.7E+07 | 2.7E+07 | 810 | 14.5339 | 13 | 0.00467 | 0.01025 |
| DMR_32 | 2 | 2.2E+08 | 2.2E+08 | 352 | 9.52427 | 7 | 0.01077 | 0.03324 |
| DMR_34 | 5 | 1.4E+08 | 1.4E+08 | 920 | 13.0068 | 13 | 0.00589 | 0.01435 |
| DMR_35 | 6 | 3.2E+07 | 3.2E+07 | 691 | 14.2228 | 12 | 0.00548 | 0.01085 |
| DMR_36 | 19 | 4.7E+07 | 4.7E+07 | 658 | 13.9082 | 12 | 0.00586 | 0.01158 |
| DMR_37 | 19 | 5.8E+07 | 5.8E+07 | 332 | 12.8219 | 10 | 0.00699 | 0.01505 |
| DMR_38 | 10 | 4.3E+07 | 4.3E+07 | 718 | 11.422 | 9 | 0.00944 | 0.02054 |
| DMR_39 | 11 | 7.3E+07 | 7.3E+07 | 377 | 10.1578 | 8 | 0.01211 | 0.02845 |
| DMR_40 | 16 | 6.8E+07 | 6.8E+07 | 388 | 8.97592 | 7 | 0.01409 | 0.03844 |
| DMR_41 | 2 | 1.6E+08 | 1.6E+08 | 841 | 11.3583 | 9 | 0.00964 | 0.02082 |
| DMR_42 | 6 | 3.3E+07 | 3.3E+07 | 285 | 10.5976 | 13 | 0.0079 | 0.02531 |
| DMR_43 | 22 | 3.8E+07 | 3.8E+07 | 564 | 12.2997 | 12 | 0.00809 | 0.01674 |
| DMR_44 | 16 | 5.8E+07 | 5.8E+07 | 564 | 12.4128 | 10 | 0.00789 | 0.01631 |
| DMR_45 | 2 | 2.8E+07 | 2.8E+07 | 793 | 12.386 | 10 | 0.00798 | 0.01638 |
| DMR_46 | 8 | 2.2E+07 | 2.2E+07 | 642 | 11.0872 | 9 | 0.01077 | 0.02232 |
| DMR_47 | 8 | 6420242 | 6421171 | 929 | 11.0146 | 9 | 0.01096 | 0.02267 |
| DMR_48 | 19 | 1.8E+07 | 1.8E+07 | 421 | 8.71938 | 7 | 0.01613 | 0.04128 |
| DMR_49 | 19 | 1.5E+07 | 1.5E+07 | 397 | 11.969 | 10 | 0.00895 | 0.01825 |
| DMR_50 | 12 | 6745057 | 6745707 | 650 | 10.7415 | 12 | 0.0104 | 0.02442 |
| DMR_51 | 8 | 1.7E+07 | 1.7E+07 | 489 | 10.6421 | 9 | 0.01235 | 0.02508 |
| DMR_52 | 6 | 7.4E+07 | 7.4E+07 | 711 | 10.5932 | 9 | 0.01257 | 0.02535 |
| DMR_53 | 2 | 286830 | 287395 | 565 | 9.46162 | 8 | 0.01612 | 0.03382 |
| DMR_54 | 8 | 1E+08 | 1E+08 | 685 | 11.4545 | 10 | 0.01036 | 0.0203 |
| DMR_55 | 7 | 1.4E+08 | 1.4E+08 | 558 | 11.3868 | 10 | 0.01064 | 0.02065 |
| DMR_56 | 2 | 2.8E+07 | 2.8E+07 | 768 | 12.1009 | 11 | 0.00923 | 0.01766 |
| DMR_57 | 7 | 9.4E+07 | 9.4E+07 | 517 | 9.2441 | 8 | 0.01774 | 0.03567 |
| DMR_58 | 1 | 1.6E+08 | 1.6E+08 | 232 | 8.01693 | 7 | 0.02288 | 0.0498 |
| DMR_59 | 6 | 656504 | 657363 | 859 | 11.8543 | 11 | 0.0097 | 0.01864 |
| DMR_60 | 3 | 1.1E+08 | 1.1E+08 | 775 | 10.9938 | 11 | 0.01181 | 0.02281 |
| DMR_61 | 13 | 3.6E+07 | 3.6E+07 | 551 | 10.652 | 11 | 0.01267 | 0.02503 |
| DMR_62 | 2 | 2.2E+08 | 2.2E+08 | 876 | 9.85404 | 9 | 0.01661 | 0.0306 |
| DMR_63 | 1 | 2.5E+08 | 2.5E+08 | 658 | 8.83477 | 8 | 0.0214 | 0.04001 |
| DMR_64 | 1 | 4.1E+07 | 4.1E+07 | 628 | 8.82649 | 8 | 0.02149 | 0.04012 |
| DMR_65 | 1 | 2.2E+08 | 2.2E+08 | 875 | 10.5723 | 11 | 0.01286 | 0.02546 |
| DMR_66 | 9 | 1.2E+08 | 1.2E+08 | 871 | 8.68161 | 8 | 0.02291 | 0.0416 |
| DMR_67 | 13 | 3.1E+07 | 3.1E+07 | 583 | 8.66934 | 8 | 0.02298 | 0.0417 |
| DMR_68 | 17 | 4.9E+07 | 4.9E+07 | 356 | 10.0408 | 11 | 0.01416 | 0.02919 |
| DMR_69 | 1 | 2.8E+07 | 2.8E+07 | 294 | 9.80104 | 11 | 0.01475 | 0.03096 |
| DMR_70 | 8 | 6.7E+07 | 6.7E+07 | 477 | 9.50929 | 9 | 0.01862 | 0.03332 |
| DMR_71 | 3 | 1.4E+07 | 1.4E+07 | 669 | 8.87056 | 11 | 0.0168 | 0.0396 |
| DMR_72 | 16 | 8.5E+07 | 8.5E+07 | 762 | 8.48372 | 8 | 0.02501 | 0.04389 |
| DMR_73 | 1 | 6.8E+07 | 6.8E+07 | 731 | 8.2944 | 8 | 0.0273 | 0.04622 |
| DMR_74 | 6 | 5E+07 | 5E+07 | 564 | 10.2 | 10 | 0.01528 | 0.02811 |
| DMR_75 | 19 | 5.6E+07 | 5.6E+07 | 547 | 10.1113 | 10 | 0.01567 | 0.02872 |
| DMR_76 | 21 | 3.6E+07 | 3.6E+07 | 730 | 10.0451 | 10 | 0.01595 | 0.02917 |
| DMR_77 | 17 | 6899207 | 6899758 | 551 | 9.85385 | 10 | 0.01669 | 0.03061 |
| DMR_78 | 6 | 3.1E+07 | 3.1E+07 | 323 | 9.25461 | 9 | 0.02039 | 0.03555 |
| DMR_79 | 6 | 2.6E+07 | 2.6E+07 | 490 | 9.06885 | 9 | 0.02184 | 0.03742 |
| DMR_80 | 5 | 1.3E+08 | 1.3E+08 | 482 | 8.78826 | 9 | 0.02396 | 0.04063 |
| DMR_81 | 2 | 1.1E+08 | 1.1E+08 | 619 | 8.232 | 8 | 0.02801 | 0.04699 |
| DMR_82 | 1 | 5.5E+07 | 5.5E+07 | 247 | 8.11761 | 8 | 0.02947 | 0.04859 |
| DMR_83 | 3 | 1.5E+08 | 1.5E+08 | 1278 | 9.45876 | 10 | 0.01842 | 0.03385 |
| DMR_84 | 12 | 5.2E+07 | 5.2E+07 | 515 | 8.87907 | 10 | 0.02098 | 0.03944 |
| DMR_85 | 17 | 3.8E+07 | 3.8E+07 | 775 | 8.7961 | 10 | 0.02135 | 0.04054 |
| DMR_86 | 15 | 7.4E+07 | 7.4E+07 | 547 | 8.36942 | 10 | 0.023 | 0.04519 |
| DMR_87 | 11 | 4.7E+07 | 4.7E+07 | 386 | 8.27875 | 10 | 0.02326 | 0.0464 |
| DMR_88 | 19 | 5.7E+07 | 5.7E+07 | 525 | 8.04511 | 9 | 0.02941 | 0.0495 |

Chr: chromosome; start: start of the DMR; end: end of DMR; width: length of DMR; area: area of DMR; L: number of annotated CpGs (p-value <0.05). DMRs were identified with a minimum of 7 annotated CpGs.

### Table S5. Differential diagnosis for affected individuals reported in this study.

| **Differential Diagnosis (OMIM)** | **Gene name (OMIM)** | **Clinical features** | **Inheritance** |
| --- | --- | --- | --- |
| CHARGE syndrome (OMIM 214800), | *CHD7* (608892) | Coloboma, heart defects, choanal atresia, mental retardation, genital hypoplasia and ear anomalies | Autosomal Dominant |
| Branchio-Oto-Renal syndrome (BORS, OMIM 113650) | *EYA1* (OMIM 601653) | Hearing loss, auricular malformations, branchial arch abnormalities and renal disease | Autosomal Dominant |
| Branchiootic syndrome 2 (OMIM 120502) | *SIX1* (OMIM 601205) | Branchial and otic anomalies | Autosomal Dominant |
| Branchiooculofacial syndrome (OMIM 113620) | *TFAP2A* (OMIM 107580) | Branchial cleft sinus defects, ocular anomalies such as microphthalmia and lacrimal duct obstruction, a dysmorphic facial appearance including cleft or pseudocleft lip/palate | Autosomal Dominant |
| Bamforth-Lazarus syndrome (OMIM 241850) | *FOXE1* (OMIM 602617) | Congenital hypothyroidism due to thyroid dysplasia, cleft palate and spiky hair, with or without choanal atresia or bifid uvula | Autosomal Recessive |

## References

1. Mark C, Jim D, Martin D, et al. *The 100,000 Genomes Project Protocol.* 2017.

2. Narasimhan VM, Hunt KA, Mason D, et al. Health and population effects of rare gene knockouts in adult humans with related parents. *Science.* 2016;352(6284):474-477.

3. Deciphering Developmental Disorders S. Prevalence and architecture of de novo mutations in developmental disorders. *Nature.* 2017;542(7642):433-438.

4. Team TRDC. R Development Core Team (2008). R: A language and environment for statistical computing.

5. Butcher DT, Cytrynbaum C, Turinsky AL, et al. CHARGE and Kabuki Syndromes: Gene-Specific DNA Methylation Signatures Identify Epigenetic Mechanisms Linking These Clinically Overlapping Conditions. *Am J Hum Genet.* 2017;100(5):773-788.

6. Aryee MJ, Jaffe AE, Corrada-Bravo H, et al. Minfi: a flexible and comprehensive Bioconductor package for the analysis of Infinium DNA methylation microarrays. *Bioinformatics.* 2014;30(10):1363-1369.

7. McCartney DL, Walker RM, Morris SW, McIntosh AM, Porteous DJ, Evans KL. Identification of polymorphic and off-target probe binding sites on the Illumina Infinium MethylationEPIC BeadChip. *Genom Data.* 2016;9:22-24.

8. Alisch RS, Barwick BG, Chopra P, et al. Age-associated DNA methylation in pediatric populations. *Genome Res.* 2012;22(4):623-632.

9. Hannum G, Guinney J, Zhao L, et al. Genome-wide methylation profiles reveal quantitative views of human aging rates. *Mol Cell.* 2013;49(2):359-367.

10. Perez RF, Santamarina P, Tejedor JR, et al. Longitudinal genome-wide DNA methylation analysis uncovers persistent early-life DNA methylation changes. *J Transl Med.* 2019;17(1):15.

11. Maksimovic J, Gordon L, Oshlack A. SWAN: Subset-quantile Within Array Normalization for Illumina Infinium HumanMethylation450 BeadChips. *Genome Biol.* 2012;13(6).

12. Badalato L, Farhan SM, Dilliott AA, et al. KMT2D p.Gln3575His segregating in a family with autosomal dominant choanal atresia strengthens the Kabuki/CHARGE connection. *Am J Med Genet A.* 2017;173(1):183-189.
